# Waste-Derived Sustainable Nanomaterials: Comprehensive Review of Synthesis Advances, Applications and Translational Challenges

**DOI:** 10.3390/nano16130792

**Published:** 2026-06-25

**Authors:** Mahima Yadav, Jason Hodge, Terrence J. Piva, Moshi Geso, Rod Lynch, Faiza Basheer, William Patterson, Alison Chapman, Rasika M. Samarasinghe

**Affiliations:** 1 School of Medicine, Faculty of Health, Deakin University, Geelong 3220, Australia; s222631596@deakin.edu.au (M.Y.); faiza.basheer@deakin.edu.au (F.B.); 2 Deakin Institute for Mental and Physical Health and Clinical Translation, Deakin University, Geelong 3220, Australia; 3 University Hospital Geelong, Geelong 3220, Australia; jason.hodge@barwonhealth.org.au (J.H.); rod.lynch@barwonhealth.org.au (R.L.); 4 School of Health and Biomedical Science, RMIT University, Bundoora 3083, Australia; terry.piva@rmit.edu.au (T.J.P.); moshi.geso@rmit.edu.au (M.G.); 5 ICON Healthcare, Epworth Geelong Hospital, Geelong 3216, Australia; billpatt@tpg.com.au (W.P.); alison.chapman@icon.team (A.C.)

**Keywords:** waste-derived nanoparticles, circular economy, sustainable nanomaterials, resource recovery, nanomaterial synthesis

## Abstract

Waste management presents a major environmental and public health challenge, creating an urgent need for strategies that convert discarded materials into higher-value products. Waste-derived nanoparticles (WDNPs) have gained increasing attention because they integrate waste valorization with the production of functional nanomaterials for environmental, biomedical, agricultural, packaging, sensing, catalytic and energy-related applications. This review critically evaluates WDNP synthesis from five major waste streams, including agricultural residues, animal-derived waste, plastic waste, electronic waste and industrial by-products. Across these categories, precursor composition strongly influences nanoparticle size, morphology, surface chemistry, stability and functional performance, making feedstock selection and processing conditions central to reproducible production. Evidence from recent studies indicates that WDNPs have broad functional potential across environmental remediation, biomedical delivery, antimicrobial systems, sustainable packaging, agriculture, energy storage and catalysis. However, translation beyond laboratory-scale studies remains limited by feedstock variability, limited reproducibility, complex purification requirements, potential toxicity, insufficient standardization and limited pilot-scale validation. By comparing synthesis approaches, application outcomes and translational barriers across waste categories, this review provides a critical overview of the opportunities and limitations of WDNPs and identifies the key requirements for their responsible development within a circular-economy framework.

## 1. Introduction

The rapid acceleration of industrialization, urbanization, and consumer-driven economies has resulted in an unprecedented accumulation of waste across multiple sectors, including agricultural residues, electronic waste, plastics, industrial by-products, and animal-derived materials [1]. These waste streams represent a major source of environmental pollution and pose significant risks to human health. Without robust, well-implemented and enforceable solid waste management strategies, these challenges are likely to intensify, resulting in long-term environmental degradation and adverse health outcomes [2]. Data from the United Nations Environment Program revealed that 11.2 billion tons of solid waste are generated globally each year, contributing substantially to environmental degradation and public health challenges [3]. Rapid urbanization, industrial growth and rising consumption have driven annual global solid waste generation, with current projections indicating that by 2050, municipal solid global waste generation will rise by nearly 70% relative to 2016 levels, reaching an estimated 3.5 billion tons per year [4]. While high-income countries discard only about 2% of generated waste, approximately 93% of waste in low and middle-income countries is either openly discarded or incinerated, creating severe environmental contamination and public health risk. The Agency for Toxic Substances and Disease Registry has highlighted severe health hazards linked with exposure to hazardous waste, including birth defects, reproductive disorders, cancers, immune dysfunction, kidney and liver damage, respiratory disease, and neurotoxicity [5]. Numerous studies further demonstrate the correlation between exposure to neurotoxic pollutants, such as heavy metals and particulate matter, and neurological impairments, including cognitive decline, anxiety, and depression across all age groups [6]. These risks underscore the urgent need for waste management technologies that not only reduce environmental contamination but also minimize human exposure to hazardous substances.

Valorization of waste into high-value products therefore represents a critical shift from linear “take-make-dispose” models toward a circular bioeconomy. Among emerging strategies, the conversion of diverse waste streams (agricultural residues, animal by-products, plastics, electronic waste and industrial effluents) into nanomaterials has gained momentum. Waste-derived nanoparticles (WDNPs) simultaneously address waste accumulation and the growing demand for sustainable nanoscale materials in environmental remediation, catalysis, sensing and nanobiotechnology [7,8]. While conventional physical and chemical nanoparticle (NP) synthesis routes offer high purity and morphological control, they are energy intensive, rely on toxic reagents and generate hazardous by-products, which are limitations that undermine their environmental credentials. Notably, biological (green) synthesis routes operate under milder conditions, incorporate natural capping agents and align directly with green chemistry and circular-economy principles. These routes have also been reported to reduce energy consumption by approximately 30% and production costs by up to 40%, while increasing production output by around 50% compared to conventional synthesis methods [9]. Despite these advances, WDNP synthesis and the transition from laboratory proof-of-concept to industrial production remains challenging due to variability in waste composition, limited reproducibility, challenges in purification and standardization, potential toxicity associated with residual contaminants, and uncertainties surrounding long-term environmental impacts. Moreover, studies report promising performance under controlled conditions but lack systematic comparison with conventionally synthesized NPs, making it difficult to assess the true viability of WDNPs in real-world applications [10].

This review provides a comprehensive and critical assessment of the synthesis, properties, and applications of NPs derived from diverse waste streams, including agricultural, animal, plastic, electronic, and industrial sources. In addition, it summarizes recent advances, giving particular attention to quantitative performance comparisons against conventionally synthesized counterparts, identification of key bottlenecks, and the integration of biotechnological advances (such as metabolic engineering and bioreactor optimization) that are essential for industrial translation. Where available, NP recovery yields from waste feedstocks have been incorporated and critically discussed, as yield represents an important parameter influencing process efficiency and economic feasibility. However, the review also highlights a major limitation identified, which is the inconsistent reporting of yield data in many of the studies, which currently limits cross-study comparisons and robust techno-economic evaluation. By highlighting these knowledge gaps and trade-offs, this review provides a balanced assessment of the opportunities and challenges associated with advancing waste-derived nanomaterials within a circular-economy framework.

## 2. Nanoparticle Synthesis Methods: Conventional to Waste-Derived Strategies

**Figure 1:**
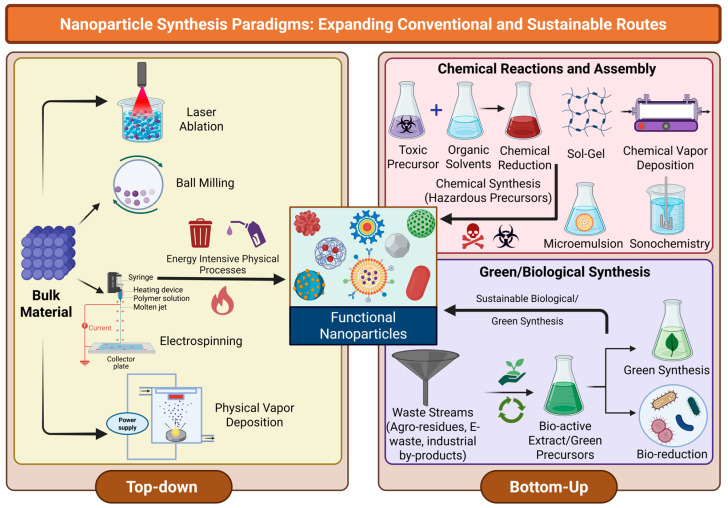
Overview of NP synthesis strategies, illustrating top-down and bottom-up approaches and their adaptation in WDNP synthesis. Biological (green), physicochemical, and thermochemical pathways highlight the growing shift toward sustainable and waste-based routes. Created in BioRender. Yadav, M. (2026) https://BioRender.com/6x41j29 (accessed on 19 May 2026).

Nanofabrication strategies are fundamentally categorized into three methodological processes: physical, chemical, and biological (green) synthesis. These methods are further organized under two operational approaches, top-down and bottom-up, based on whether nanostructures are produced by fragmenting bulk materials or assembled from smaller building blocks using chemical and biological methods [11] (Figure 1). Top-down fabrication involves the physical or mechanical breakdown of larger materials into nanoscale particles using physical techniques which can offer precise control over the structure and size of particles. This approach offers advantages like uniform NP size and shape and production of large quantities, which are typically energy intensive and costly [12]. In contrast, bottom-up fabrication assembles nanostructures from atomic or molecular building blocks through self-organization and chemical reactions. While bottom-up fabrication benefits from self-assembly to achieve finer material compatibility and nanoscale resolution with fewer processing steps, its dependence on purified precursors and hazardous reagents remains a notable sustainability drawback [13]. Recent advancements have introduced hybrid technologies that combine elements of both top-down and bottom-up approaches; these include nano-molding [14], directed self-assembly [15] and DNA-enabled nanofabrication [16]. While these technologies offer enhanced structural control and functionality, they remain largely dependent on pristine materials and complex processing and thus have limited applicability for WDNP synthesis.

A critical analysis of conventional nanofabrication models reveals a fundamental challenge between technical precision and environmental sustainability. Physical routes, while offering high-purity, chemically-free NPs, are intrinsically constrained by high energy demands and expensive infrastructure, which often fail to satisfy the requirements for low-cost, high-volume production. Conversely, chemical bottom-up strategies provide precise stoichiometric and morphological control but are significantly limited by the environmental and economic issues associated with toxic solvent disposal and hazardous precursor requirements. This dual nature of conventional synthesis approaches, wherein physical methods sacrifice energy for purity and chemical routes sacrifice environmental safety for structural precision, underscores a significant gap in sustainability. Consequently, biological (green) synthesis has gained considerable traction as a sustainable alternative, though conventional methods remain relevant where precise morphological control and batch-to-batch reproducibility are required [11].

Biological synthesis, commonly referred to as green synthesis, represents environmentally friendly processes for NP synthesis that utilize natural biological resources as an alternative to conventional physical and chemical methodologies. These biogenic approaches offer distinct advantages, including improved biocompatibility, enhanced surface functionality and stability, reduced reliance on hazardous chemicals, lower energy requirements, and cost effectiveness [17]. By operating under milder temperatures and pressures, green synthesis avoids toxic reagents and minimizes the energy-intensive demands of traditional routes while producing NPs with favorable physicochemical characteristics and enhanced functional stability, making them inherently safer for environmental and biomedical applications. As a result, green synthesized NPs are increasingly regarded as sustainable alternatives that align with green chemistry principles, positioning them as cornerstones for achieving sustainable development goals (SDGs) [18]. Green synthesis employs a wide range of biological systems including yeast, fungi, bacteria, algae and plant extracts (flowers, leaves, fruits, seeds, roots, peels). These biological entities contain diverse biomolecules, such as phenolics, proteins, polysaccharides, enzymes, and secondary metabolites, that act as electron donors, reducing metal ions to their elemental or oxide forms. Furthermore, functional groups such as hydroxyl, carboxyl, amine, and carbonyl functional groups bind to NP surfaces, providing intrinsic capping and stabilization that prevents agglomeration and enhances stability of the resulting NPs [9]. This review encompasses the full range of synthesis approaches used to convert waste streams into functional nanomaterials, including thermochemical, physicochemical, hydrometallurgical and biological routes. The term “green synthesis” is applied specifically to biological and plant-mediated routes where explicitly mentioned. Table 1 presents a comparative overview of the major physical, chemical, and biological synthesis methods, emphasizing their underlying principles, key advantages, limitations and applications.

## 3. Waste Sources for the Synthesis of Nanoparticles

Waste extracts obtained from diverse sources, including agricultural residues, industrial by-products, e-waste, and municipal solid waste, represent a rapidly expanding and strategically important resource for the sustainable synthesis of NPs [3]. These waste-derived extracts are fundamentally enriched with bioactive secondary metabolites such as polyphenols, proteins, polysaccharides and reducing sugars, which simultaneously serve as endogenous reducing and steric stabilizing (capping) agents during the nucleation phase [35]. These biogenic products have broad applicability across multiple industries including the healthcare sector for drug delivery, diagnostic platforms and therapeutic agents, the food industry for nutritional enhancement, smart packaging and preservation technologies and cosmetics for active ingredients in skincare, anti-ageing formulations and UV protection [36].

**Figure 2:**
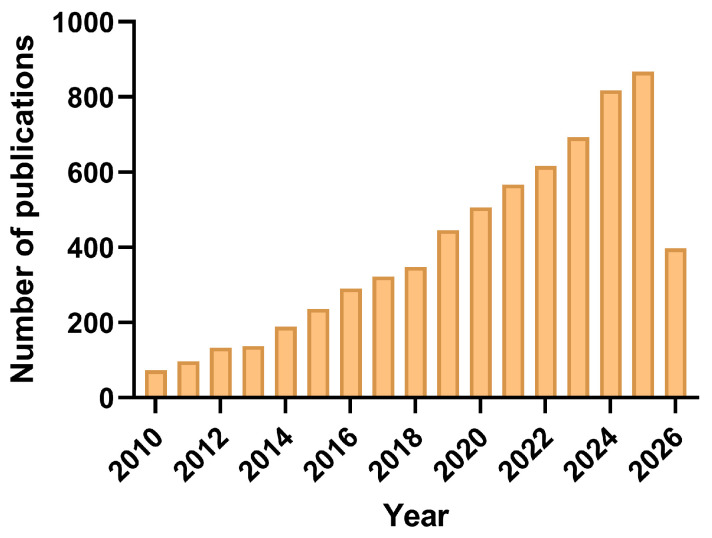
Annual distribution of publications on waste-derived NPs retrieved from the PubMed database (as of June 2026), using the search query “waste” AND “nanoparticles”.

The generation of waste materials has increased exponentially due to rapid industrialization, population growth, and technological advancement, which has exacerbated environmental degradation and contributes significantly to climate change. As illustrated in Figure 2, the number of scientific publications on WDNPs has increased significantly over the past decade, with notable peaks between 2022 and 2025. While this trend reflects growing scientific interest, it also highlights a critical transition in the field, from proof-of-concept studies toward application-driven and scalable nanomaterial development. The conversion of waste into functional nanomaterials therefore represents not only a sustainable solution, but also an emerging technological paradigm within materials science [37]. However, converting heterogeneous waste into reproducible, high-performance nanomaterials remains challenging due to variable composition, scalability barriers, and limited techno-economic validation. Standard protocols typically involve collection, pre-treatment, extraction, and controlled synthesis, yet real-world translation is hindered by batch-to-batch inconsistency and downstream purification demands [38]. Therefore, their performance must be critically evaluated in the context of real-world applicability, rather than isolated laboratory conditions. The following sections provide a systematic and critical analysis of major waste streams used in NP synthesis, highlighting both their potential and inherent limitations.

### 3.1. Agricultural Waste-Derived Nanoparticles

**Figure 3:**
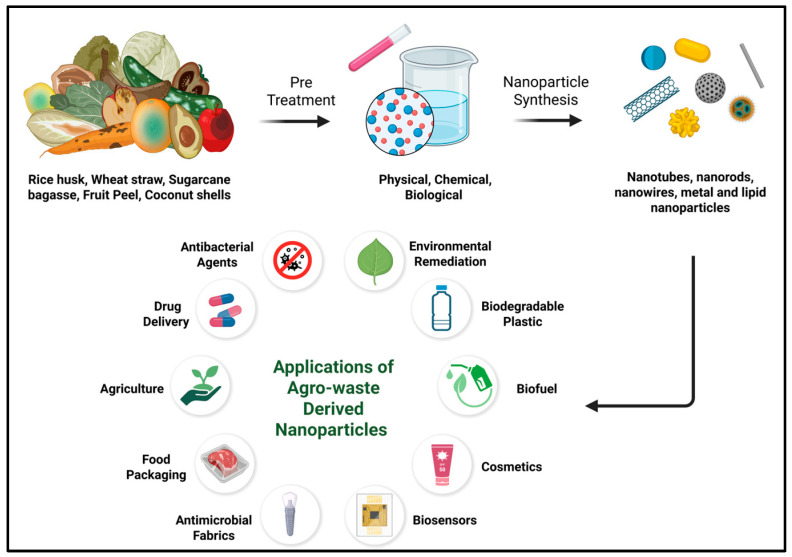
Synthesis of NPs from agricultural waste and their diverse applications. Created in BioRender. Yadav, M. (2026) https://BioRender.com/s2lz16c (accessed on 19 May 2026).

Agricultural waste streams, including horticultural, aquacultural, and domestic kitchen by-products (e.g., rice husks and straw, sugarcane bagasse, bamboo leaves, banana peels, and coconut husks) represent one of the largest and most accessible waste streams, generating ~2.5 billion tons of biomass annually [39]. These materials are rich in valuable biopolymers and phytochemicals such as lignin, cellulose, chitin, hemicellulose, polyphenols, carotenoids and essential oils, offering a sustainable, low-cost platform for WDNP synthesis while addressing landfill burden, greenhouse gas emissions, and resource depletion [35,38]. The effective valorization of these low-quality biomass resources therefore represents both an environmental necessity and an economic opportunity, aligning strongly with the “waste-to-wealth” concept [40]. Within this context, technological advances in NP fabrication from agricultural residues have attracted significant attention due to their potential to generate high-value nanomaterials for diverse applications, particularly in the area of food packaging [41], environmental remediation [42], biomedicine [43], bioplastic [44], biofuel [45], supercapacitor performance [46] and in energy storage for enhancing battery capacity [47], as illustrated in Figure 3. However, despite this versatility, the performance of agricultural WDNPs is highly dependent on feedstock composition, which varies significantly with plant species, geographical origin, and processing conditions, introducing challenges in reproducibility and standardization.

#### 3.1.1. Sustainable Food Systems

In the food sector, agro-industrial waste has emerged as a promising feedstock for the development of eco-friendly packaging materials or coatings that can serve as sustainable long-term alternatives to petroleum-based plastic packaging systems. These nanocomposites extend shelf life of food products by blocking ultraviolet (UV) radiation, reducing oxygen consumption, and exhibiting localized antimicrobial activity to inhibit food spoilage [41]. The fabrication process typically involves the deconstruction of complex biomass into functional components, followed by extraction of fibers and polymers suitable for material synthesis [48]. Concurrently, inherent phytochemicals present within the wastes act as natural stabilizing and reducing agents that react with metal salts, resulting in the green synthesis of different NPs that are subsequently incorporated with film-forming polymer matrices such as PVA, PLA, starch, gelatin, chitosan, and cellulose [49,50]. To enhance flexibility and processability, these polymers are often dissolved in aqueous media or plasticizers such as glycerol. The resulting NP–polymer combinations are cast into a flat mold, uniformly distributed with a blade to form a homogeneous film, dried, peeled off, and processed into suitable forms for food packaging, such as wraps, bags, and trays [51].

A representative example of this application was reported by Dey et al. [52], who utilized solvent casting to fabricate PVA-based nanocomposite films containing cellulose nanocrystals (CNCs, 377 ± 21.5 nm) and chitosan NPs (CNPs, 291 ± 4.6 nm) derived from mango peel by-products. The resulting films exhibited enhanced mechanical strength, exceptional thermal stability up to 430 °C and antifungal activity achieving approximately 70% inhibition of *Colletotrichum gloeosporioides.* Notably, these films demonstrated biodegradability of up to 88% in dry soil, and effectively extended mango shelf life by preventing post-harvest decay over a 20-day storage period [52]. Similarly, Daassi et al. [53] optimized the electrospray production of lignin NPs from rice husk using response surface methodology. Under optimal parameters (49.1 mg/mL lignin, 0.5 mL/h flow rate, 25.4 kV voltage, and 22 cm tip-to-collector distance), uniform spherical NPs (260 ± 10 nm), with low polydispersity index (PDI = 0.257) and high colloidal stability (zeta potential (ZP) of −35.2 ± 4.1 mV) were obtained. The study reported high rice husk organosolv lignin purity (96%), although the final LNP product yield/recovery was not stated. When incorporated into PLA films, particularly as PLA-grafted LNPs, these nanocomposites enhanced UV-blocking capacity (UV transmittance decreased from 58.7% to 1.10%)—a four-fold increase in elongation at break, and more than a twelve-fold enhancement in antioxidant activity [53]. Furthermore, AgNPs synthesized using aqueous extracts of pomegranate and kinnow fruit peels, which acted as reducing and capping agents, and when applied to cellulose-based films significantly improved their antioxidant and antimicrobial properties, reduced oxygen and moisture permeability, and extended the shelf life of packaged bread, providing a biodegradable and sustainable alternative to conventional polyethylene-based food packaging material [54]. Although the study reported peel extract yields of 23% for pomegranate and 18.5% for kinnow, the final AgNP product yield/recovery was not stated.

Beyond active food packaging, agricultural WDNPs have attracted growing interest in functional food and nutraceutical delivery applications. Wu et al. [55] prepared cellulose nanocrystals (CNCs), cellulose nanofibers (CNFs), lignin-containing cellulose nanocrystals (LCNCs), and lignin-containing cellulose nanofibers (LCNFs) from corn stover for Pickering emulsion stabilization and quercetin delivery. The authors reported on the chemical composition of the nanocellulose fractions, including retained lignin contents of 25.7% in LCNCs and 22.9% in LCNFs, but the final product yield/recovery of these fractions was not stated. Among these, lignin-containing nanocellulose conferred enhanced ultraviolet protection, emulsion stability, and quercetin bioaccessibility, underscoring the utility of crop residues as sustainable food-grade nanocarriers [55]. In a related approach, Golub et al. [56] synthesized selenium NPs using tomato pomace-derived pectin as a stabilizer, functionalized with polyphenol-rich extracts from olive pomace, mandarin peel, and grape seed. The resulting NPs showed improved biocompatibility relative to inorganic selenite, alongside enhanced antioxidant activity and stability under simulated gastrointestinal digestion, supporting their potential as sustainable nutraceutical nanoformulations [56], with product yield/recovery not specified.

#### 3.1.2. Environmental Remediation

In environmental remediation, agricultural WDNPs have shown substantial promise in various applications, particularly for water purification and pollutant removal [42]. For instance, a novel green synthesis approach using polyphenol-rich onion peel extract was used to synthesize green nano-zero valent iron (G-NZVI) particles with sizes ranging from 100–600 nm. These particles achieved complete removal of 50 mg/L bromate (BrO_3_^−^) within 2 min under both aerobic and anaerobic conditions, outperforming conventional reductants due to enhanced reactivity and oxidation resistance, thereby demonstrating the effectiveness of onion peel waste as a sustainable resource for water treatment applications [57]. In another study, rice husk-derived nanoscale biochar produced via pyrolysis and mechanical grinding was employed as an inexpensive, environmentally friendly adsorbent for fluoride (F^−^) removal from groundwater. The study reported a biochar yield of 41.66% from rice husk biomass. The material achieved 90% fluoride removal using only 1 g/L absorbent within 60 min of contact time. Sorption behavior followed both Freundlich (R^2^ = 0.995) and Langmuir (R^2^ = 0.991) isotherms, with pseudo-second-order kinetics, indicating strong adsorption capacity and applicability under neutral pH conditions [58]. Despite these promising results, long-term regeneration, disposal, and potential secondary pollution require further evaluation.

Rice straw has been investigated as a precursor for both photocatalytic and nutrient-recovery NPs. Kamboj et al. [59] synthesized silica NPs from rice straw, reporting notable dye degradation capacity and antibacterial activity, with proposed application in water remediation [59]; however, SiNP product yield/recovery was not specified. Priya et al. [60] took a different approach, extracting cellulose nanofibers from rice straw and combining them with carboxymethyl cellulose and Fe(OH)_3_ NPs to fabricate hydrogel beads for phosphate recovery from wastewater. This study reported cellulose nanofibers yields of 43% from raw fibers and 33% from extracted cellulose fibers, while the final hydrogel bead yield/recovery was not reported. The phosphate-loaded beads were subsequently repurposed as slow-release fertilizers, effectively bridging wastewater treatment with nutrient recycling and agricultural reuse [60].

#### 3.1.3. Energy and Bio-Functional Systems

Within a circular-economy framework, WDNPs are being repurposed for advanced functional applications including biosensing, biofuels, bioplastics, and energy storage, transforming low-value residues into high-performance technological materials. These applications exploit the tunable physicochemical properties of WDNPs, such as high surface area, surface functionality, porosity, and electrical conductivity, enabling both improved performance and enhanced sustainability. In the field of environmental monitoring and biosensing, WDNPs have shown considerable promise. For instance, Alfi et al. [61] produced nitrogen-doped carbon dots (NCDs, 5–8 nm) from sugarcane bagasse and ammonium hydroxide (NH_4_OH) as a passivating agent via a one-pot hydrothermal technique. The resulting NCDs exhibited strong blue fluorescence with an emission peak at 447 nm and a quantum yield of 24.81%, and when embedded in cellulose paper, the NCDs formed a low-cost dipstick sensor for tetracycline detection (0.01–150 μM) used in food safety monitoring [61].

Another major advance is the development of sustainable bioplastics as alternatives to petroleum-based polymers. Given that only approximately one-third (33%) of the 350–380 million tons of plastic generated each year is recycled, with the remainder discarded leading to environmental harm through microplastics and chemical leaching, biodegradable polymers derived from renewable natural sources, such as starch, cellulose, and proteins, have gained increasing attention as sustainable alternatives to fossil-based plastics directly contributing to SDG 12 (Responsible Production). A recent study addressed the environmental concerns associated with edible feedstocks by utilizing agricultural and household wastes, such as starch from sweet potato peels, cellulose from banana pseudo-stems, and glycerol from used cooking oil, as raw materials for bioplastic production. They demonstrated that these materials exhibited excellent physicochemical, mechanical, and biodegradable qualities, with an optimized cellulose content of 30% (*w*/*w*), which made it ideal for use in food packaging applications. This approach not only reduced plastic pollution but also aligned with sustainable development goals including SDG 3 (health), SDG 12 (responsible production), and SDG 8 (economic growth) [62].

In addition, agricultural WDNPs have also been investigated for sustainable biofuel production and as high-performance energy storage systems. With high-performance energy storage systems, tea saponin (TS), an abundant agricultural by-product, was chemically activated with potassium hydroxide (KOH) (1:1 mass ratio) to produce porous biochar with a high surface area (1550.03 m^2^/g), large pore volume (0.9076 cm^3^/g), and high levels of functional groups. When evaluated as a supercapacitor electrode, the optimized biochar exhibited excellent electrochemical performance, including a specific capacitance of 278 F/g, an energy density of 27.01 Wh/kg, and 100% capacitance retention over 10,000 charge–discharge cycles in an organic electrolyte [63]. Another promising example involves activated carbon derived from sugarcane bagasse, where KOH-activated bagasse carbon achieved a specific capacitance of ~253.41 F/g at 0.5 A/g, and symmetric devices constructed from these electrodes delivered an energy density of ~17.91 Wh/kg and power density up to ~2990 W/kg, with ~93.9% capacitance retention after 10,000 cycles [64]. These studies illustrate that agricultural WDNPs can meet performance requirements for practical supercapacitor applications, highlighting their potential as sustainable alternatives to conventional electrode materials.

Carbon NPs derived from agricultural waste have found application in solar thermal conversion and hydrogen production. Suraj et al. [65] developed a coffee husk char-derived carbon-based nanofluid for solar thermal applications. The nanofluid contained near-spherical carbon NPs with an average particle size of 6.24 nm, and showed a zeta potential of −59.4 mV, thermal conductivity of 0.87 W/mK, and a solar-weighted absorption fraction of 62.6%. It demonstrated higher solar absorption and photothermal conversion efficiency than deionized water, with a maximum photothermal conversion efficiency improvement of 14.35%, positioning coffee husk-derived carbon-based nanofluids as low-cost working-fluid candidates for solar thermal systems; however, product yield/recovery was not reported [65]. In the context of hydrogen generation, Ruiz López et al. [66] developed cobalt-modified catalysts supported on corn husk-derived biochar for NaBH_4_ hydrolysis. The best-performing BP400 catalyst contained highly dispersed amorphous CoO/Co_3_O_4_ NPs of approximately 2.1 nm on the biochar surface and achieved a hydrogen generation rate of 1416.5 mL g^−1^ min^−1^, with an activation energy of 45.13 kJ mol^−1^. The BP400 catalyst was produced with a reported biochar yield of 36.1%, supporting the potential of corn husk waste as a sustainable precursor for hydrogen-generation catalysts [66]. These findings suggest that agricultural waste-derived carbon can serve as both a nanocarbon precursor and a catalytic support, though long-term stability, metal leaching, and catalyst regeneration remain key challenges [66].

#### 3.1.4. Agricultural Systems

Agricultural WDNPs are increasingly being explored as nano-enabled slow-release fertilizers and biopesticides, due to their ability to deliver nutrients in a controlled and sustained manner. By acting as transporters for nutrients or active chemicals, these NPs enable controlled nutrient release, reduce leaching and volatilization losses, and minimize environmental contamination. By reducing nitrogen loss from leaching and volatilization, slow-release fertilizers increase nutrient usage efficiency and lower pollution levels in the environment [67]. In one such approach, nanostructured date palm pits (nDPPs) produced via planetary ball milling were combined with MgO and monopotassium phosphate (KH_2_PO_4_) in an optimized ratio to create a novel nano-enabled fertilizer (NEF). This NEF-treated soil improved water retention by 5.6 times more compared to conventional fertilizer, which greatly enhanced soil moisture. In addition, compared to the conventional fertilizers, NEF also provided a regulated, prolonged release of phosphorus (P, 22.4%), K (35.7%), and Mg (47%) with lower nutrient leaching losses (8.9% P, 2.9% K, 16.9% Mg). Pot experiments confirmed increased maize biomass and nutrient uptake, demonstrating the agronomic benefits of this approach [68].

Silica-rich agricultural residues have been converted into nanosilica materials with plant growth-promoting properties. Bekkam et al. [69] synthesized rice husk-derived nanosilica with high purity of 99% and a reported recovery of 18%. The nanosilica showed spherical morphology, amorphous structure, siloxane bonding, and an average particle size of 69.1 ± 2.10 nm. Its application improved maize biomass, root development, photosynthetic performance, silicon uptake, and micronutrient nutrition under both irrigated and drought-stressed conditions, with optimal responses observed at 20 mg kg^−1^ under irrigated conditions and 30 mg kg^−1^ under drought stress [69]. Higher doses were associated with mild growth inhibition, indicating that dose optimization is necessary prior to field application. Kumar et al. [70] converted banana peel waste into mesoporous biogenic nanosilica, with the highest product recovery obtained under HCl-assisted heat treatment of banana peel waste ash. Product yield was calculated as recovered silica mass relative to ash mass, although the exact numerical value was presented graphically rather than stated in the text. The biogenic nanosilica was incorporated into a nano-biopriming system with *Bacillus subtilis*, resulting in improved seed germination, seedling growth, biomass accumulation, and stress tolerance markers [70]. While these findings support a role for agricultural waste-derived nanosilica in sustainable crop production, field-scale validation, long-term soil safety, and soil microbiome assessment remain areas requiring further work [69].

#### 3.1.5. Biomedical and Antimicrobial Systems

Agricultural residues have also been employed in the development of multifunctional biomedical nanocomposites. Kinoan and Katas [71] utilized spent mushroom substrate as both a cellulose source and a reducing medium to prepare AgNP-loaded TEMPO-oxidized cellulose nanofiber composites. The highest cellulose nanofiber yield was 43.21 ± 0.73% under 2% NaOH and bleaching conditions, although final AgNP/ToCNF nanocomposite yield/recovery was not reported. The resulting composites exhibited antibacterial activity against *S. aureus*, *P. aeruginosa*, and *E. coli*, with lower cytotoxicity than free AgNPs [71]. Verma et al. [72] synthesized zinc-integrated cellulose NPs from sugarcane bagasse, reporting a cellulose extraction yield of 0.56 ± 0.01 g/g dry biomass and a final cellulose NP-Zn yield of 67.8 ± 1.3%. The NPs showed antioxidant activity, low hemolysis, and biocompatibility with human embryonic kidney cells (HEK-293), alongside moderate anticancer activity against the triple-negative breast cancer cell line (MDA-MB-231), and plant root growth stimulation [72]. Though these findings point to the broader biomedical potential of agricultural residue-derived nanocellulose, in vivo validation and long-term safety assessment have yet to be established.

Nanocellulose, produced from plant materials, represents another important class of agricultural WDNPs due to its high surface area, low density, biodegradability, and mechanical durability, making it perfect for long-lasting, high-quality nanoproducts. Yahya et al. [73] demonstrated this application by extracting cellulose nanofibers (CNFs) from empty oil palm fruit bunches using a modified supercritical carbon dioxide technology, which resulted in higher yield and lower ash content compared to conventional techniques. The CNFs were subsequently processed into bioaerogel scaffolds using high-pressure homogenization and freeze-drying, with chitosan incorporated to enhance mechanical strength and water stability, and cinnamon essential oil (CEO) added to improve antibacterial functionality. The aerogels exhibited high porosity (90.8–99.1%), tunable density (8.11–141.2 mg/cm^3^), and surface area (18.7–145.3 m^2^/g), along with strong antibacterial activity against *S. aureus* and *E. coli* due to the presence of CEO. In addition, the resultant biopolymer-based aerogels demonstrated excellent compatibility and were non-toxic to L929 fibroblast cells, highlighting their potential for wound healing and skin regeneration applications [73].

Overall, agricultural WDNPs offer significant advantages, including low-cost, reduced environmental burden, intrinsic sustainability, and multifunctionality across environmental, agricultural, biomedical, and energy applications. The ability to integrate waste valorization with nanomaterial synthesis supports circular economy principles and contributes to resource efficiency. Table 2 provides an extensive overview of NPs generated from agricultural waste.

Despite the promising laboratory-scale experiments, several challenges remain that hinder large-scale implementation. These include variability in waste composition, limited control over NP size and surface chemistry, scalability constraints, and the need for standardized processing and safety assessment protocols. Additionally, energy-intensive pretreatment steps and potential secondary waste generation may offset environmental benefits if not carefully managed [39]. Most systems reviewed remain at laboratory or simulated-condition stages, and several synthesis routes involve energy-intensive or chemically harsh steps that may offset the environmental benefits of waste-derived feedstocks. Future work should prioritize reproducible synthesis, realistic application testing, long-term ecotoxicity, and life-cycle assessment. Addressing these challenges will therefore be critical for translating agricultural WDNPs from laboratory-scale to industrial and commercial production.

### 3.2. Animal Waste-Derived Nanoparticles

While microbial and plant-mediated NP synthesis have dominated the green nanotechnology landscape, animal-derived biomass remains a comparatively under-investigated resource for NP manufacturing, despite representing a chemically rich and functionally diverse feedstock. Unlike plant systems, animal waste is not only an environmental burden but also a reservoir of structurally complex biopolymers, including calcitic matrices, fibrous proteins, and lipid assemblies, that can act as both reducing agents and templating scaffolds during nanoparticle formation. The global meat and seafood industries generate tremendous quantities of waste, with a large part of the animal biomass remaining unused after processing. Quantitatively, the conversion efficiency of livestock into meat remains low, with only around 68–72% of chickens, 78% of turkeys, 52% of sheep/goats, 60–62% of pigs, and 50–54% of cows processed into consumable meat, with the remainder classified as waste [86]. Similarly, more than 65.2 million metric tons of fish waste are generated worldwide each year during seafood processing [87]. Animal waste contains vital components such as proteins (chitin, collagen, bioactive peptides, gelatine), minerals (calcium and phosphates), pigments, and essential lipids. However, improper disposal can result in hazardous microbial proliferation and elevated chemical and biological oxygen demand (COD and BOD) due to the high concentrations of biodegradable proteins, fats, and lipids present in these materials [88]. When these residues infiltrate aquatic ecosystems, microbial decomposition consumes large amounts of dissolved oxygen, resulting in oxygen depletion that can cause hypoxia or anoxia, and subsequent mass mortality of aquatic life. In addition, chemically oxidizable organic matter contributes to high COD values, reflecting increased pollutant loads that reduce water quality, promote eutrophication, and disrupt aquatic ecosystems [87].

A wide range of animal-derived wastes, including fur, feathers, poultry plumage, eggshells, fish scales, shrimp shells, cow bones, hooves, dung, and even human hair have been investigated as precursors for NP production [89]. Furthermore, unconventional animal wastes, such as cobwebs, paper wasp nests, and cockroach wings are emerging as viable templates for specialized nanofabrication. These diverse feedstocks have enabled the synthesis of a range of nanomaterials with applications across biomedicine, environmental remediation, agriculture, and energy systems. Despite the abundance and projected growth of animal biomass, the valorization and industrial scalability of animal waste remains limited compared to other systems such as plant-based or plastic waste streams. This gap comes from inconsistent feedstock quality driven by differences in diet, species, and processing conditions, which directly impacts NP reproducibility and makes standardized industrial protocols difficult to establish [90]. Nevertheless, these limitations highlight a largely untapped opportunity: with targeted research investment, animal waste valorization could become a significant and renewable feedstock for high-value nanomaterial production within a circular bioeconomy framework.

#### 3.2.1. Biomedical and Drug-Delivery Systems

The inherent biocompatibility of animal-derived materials makes them ideal candidates for clinical applications, particularly in controlled drug delivery. Avian eggshells, commonly discarded as waste by households, restaurants and bakeries, contain approximately 94% calcium carbonate (CaCO_3_), 4% magnesium carbonate (MgCO_3_), and 1% calcium phosphate (Ca_3_PO_4_) [91]. In a study by Render et al. [92], CaCO_3_ NPs were synthesized from eggshells using a top-down ball-milling method. Characterization revealed highly crystalline NPs with an approximate size range of 10 to 60 nm and highly porous architecture, which were biocompatible and non-toxic. When utilized as a core material for controlled-release tablets containing the chemotherapeutic drug 5-fluorouracil, these NPs containing tablets maintained gastrointestinal stability for up to three hours in rabbit models, indicating they were suitable for controlled and delayed drug release in a physiological system, highlighting their suitability as oral drug-delivery vehicles [92].

Marine animal waste, such as chitosan from crustacean shell and gelatin from fish skin offer a unique amino acid and polysaccharide profile for nanocarrier fabrication. Studies by Samrot et al. [93] and Subara et al. [94] explored biopolymer-based NPs derived from marine sources as alternative drug-delivery carriers. One study extracted chitosan from crab shells using varying concentrations of hydrochloric acid and employed sodium tripolyphosphate and barium chloride (BaCl_2_) as crosslinkers to form curcumin-loaded NPs (500 nm). These particles demonstrated favorable encapsulation efficiency, controlled release behavior, and significant antimicrobial activity against *Pseudomonas aeruginosa* [93]. Although this study described the extraction of chitosan from crab shells, the numerical chitosan extraction yield and final NP recovery were not reported. Complementary work synthesized gelatin NPs from tilapia fish skin using a two-step desolvation procedure, which was adjusted for particle size and encapsulated with 5-fluorouracil, showing a sustained release profile governed by Fickian diffusion kinetics [94]. Both delivery techniques exhibited antibacterial action and prolonged drug release kinetics, highly suited for biological applications. Another recent application in drug delivery involved keratin NPs synthesized from poultry feather waste. These NPs loaded with penicillin and vancomycin enhanced effectiveness against *Staphylococcus aureus* by 4-fold and 3.8-fold, respectively. Molecular docking revealed that hydrogen bonding between the amine group (Asparagine 54 in keratin) and the O13 atom of penicillin facilitated encapsulation of the β-lactam ring within the keratin structure, protecting it from enzymatic degradation and increasing its potency against microbes [95].

An emerging field that is gaining significant attention is the use of animal wastes for regenerative medicine. Animal bones provide a practical alternative for bone regenerative medicine, where organic and inorganic materials from sources such as fish bones [96] and scales [97], clam shells [98], and mammalian bones including porcine [99], bovine [100], and ostrich [101] are utilized to synthesize NPs. Fish bones from species such as silver pomfret and bluefin trevally, typically regarded as marine food industry trash, were used to successfully synthesize Ca-deficient hydroxyapatite (HAp) NPs (approximately 150 nm) via heat treatment, producing Mg-substituted HAp with enhanced biodegradability and suitability for drug delivery [102]. Senthil et al. [103] fabricated bone implants by blending Bluefin trevally-derived HAp with demineralized bone matrix and gelatin, where in vitro tests with HaCaT human keratinocyte cell lines showed high biocompatibility in MTT assays (cell viability assay), indicating the implant’s potential for bone tissue engineering [103]. A range of other animal wastes have also been successfully used to develop nanomaterials for regenerative medicine applications. Keratin extracted from chicken feather waste, with a reported extraction yield of 78.4%, has been electrospun into polymeric nanofibrous mats and used for wound healing and soft tissue regeneration applications [104]. These polyacrylonitrile (PAN) nanofibers exhibited high porosity (>80%), suitable tensile strength, and excellent cytocompatibility in the presence of keratin (0.05 wt%) compared with nanofiber without keratin. In vitro studies demonstrated enhanced antibacterial activity against *Pseudomonas aeruginosa* (30 ± 0.17 mm inhibition) and *S. aureus* (29 ± 0.31 mm inhibition zone) with PAN/0.05% keratin nanofibers. Similarly, collagen purified from tilapia fish skin waste was fabricated into dialyzed and self-assembled collagen sponges using freeze-drying, yielding highly porous, water-absorbent, and thermally stable materials. These materials demonstrated excellent cellular and blood compatibility, and in an in vivo model studying wound healing and homeostasis, the self-assembled collagen sponge showed rapid hemostasis and enhanced wound healing performance comparable to, or exceeding, that of commercial bovine collagen dressings [105].

Mineral-rich animal wastes have been investigated as precursors for hydroxyapatite-based platforms in bone regeneration. Marine shell wastes, mammalian bones, and eggshells are of particular interest, as their calcium-rich composition closely resembles the inorganic phase of native bone. Discarded oyster shells have been transformed into Mg-doped HAp micro/NPs through a one-pot, low-temperature hydrothermal process, producing cytocompatible and osteoinductive particles that supported osteogenic differentiation of mesenchymal stem cells [106]. This oyster shell-derived system reported near-complete phase transformation to HAp, with >99.5 wt% HAp achieved under optimized hydrothermal conditions; however, final recovered HAp mass or product yield was not reported. Buffalo waste bones have been used as a biogenic source of HAp NPs, where HA-700 particles with spherical bud-like morphology promoted femoral bone defect healing in rats [107]. In this study, HA phase purity was reported as 84.68% for HA-700 and 88.99% for HA-1000, but these values represent phase purity rather than synthesis yield, and final recovered HAp yield was not reported. Eggshell particles, with and without native membrane, have been incorporated into 3D-printed alginate dialdehyde–gelatin scaffolds, with improvements reported in elastic modulus, apatite formation, swelling behavior, and cytocompatibility with preosteoblast-like cells [108]. Although this scaffold study showed improved printability, mechanical strength, mineralization, biodegradation behavior, and cytocompatibility, eggshell particle recovery and final scaffold production yield were not reported. These findings support the use of shell, bone, and eggshell wastes as low-cost, sustainable precursors for HAp-based bone grafts and regenerative biomaterials.

Biopolymer-rich animal residues have also been explored as sustainable sources for antimicrobial and drug-delivery NPs. Chitin- and chitosan-rich wastes, including crustacean shells and insect exoskeletons, can be processed into nanoscale carriers with positive surface charge, bioadhesive properties, and antimicrobial activity. Black soldier fly pupal (*Hermetia illucens*) exoskeletons have been converted into nano-chitosan via sodium tripolyphosphate (STPP)-based ionic gelation, showing antimicrobial activity against both Gram-positive and Gram-negative bacteria, expanding the field beyond conventional seafood processing residues toward insect farming by-products as renewable sources of biomedical biopolymers [109]. This study reported a chitin extraction yield of 29.0 ± 0.2% from black soldier fly pupal exoskeletons, although the final nano-chitosan recovery or NP yield was not reported.

These studies show that animal WDNPs are a versatile and sustainable platform for biomedical and drug delivery. Protein and mineral wastes can be turned into biocompatible NPs with adjustable size, surface chemistry, and drug-binding capacity. These nanocarriers control and sustain drug release, and improve drug stability, bioavailability, and efficacy through protective encapsulation and interactions. However, translating them to clinical use requires thorough toxicological evaluation, batch consistency, and regulatory validation, which are still underdeveloped for animal WDNPs.

#### 3.2.2. Environmental Remediation and Agricultural Systems

Animal-derived nanomaterials have demonstrated significant potential in environmental remediation, particularly in pollutant adsorption, photocatalysis, and antimicrobial applications. Researchers Adaikalam et al. [110], Jalu et al. [111], and Hemmami et al. [112] utilized chicken eggshells, a Ca-rich bio waste, for the green synthesis of CaO NPs (generally 5–30 nm in size and with high crystallinity and surface characteristics) through high-temperature calcination without chemical reagents, due to the inherent purity of CaCO_3_. Adaikalam et al. revealed strong photocatalytic degradation of methylene blue dye (76% under sunlight in 45 min and 55% under UV in 10 min), whereas Hemmami et al. [112] demonstrated significant antibacterial and antifungal activity against *E. coli*, *S. aureus*, *K. pneumoniae*, and *C. albicans*, outperforming ampicillin in some cases. Jalu et al. further showed that eggshell-derived CaO NPs effectively adsorbed heavy metals such as Pb^2+^, Cd^2+^, Cr^2+^, and Hg^2+^ from aqueous solutions, achieving up to 99% elimination under optimal conditions (pH ~6.9, 0.838 g dosage, ~30–70 min contact time) [112]. However, these studies did not provide final CaO NP recovery or synthesis yield.

Beyond eggshell-derived CaO NPs, recent studies show that animal WDNPs can also support pollutant removal and environmental monitoring. Oyster shell-derived HAp has shown potential for water purification, with Guo et al. [113] synthesizing a porous HAp adsorbent from discarded oyster shells via pseudomorphic replacement. The resulting material showed a higher surface area than untreated oyster shell powder and improved humic acid adsorption in both batch and fixed-bed column experiments using real surface water, with potential application in reducing disinfection by-product precursors in drinking water treatment [113]. The study reported 87.6% HAp phase transformation after 48 h, but final product yield was not provided. In environmental sensing, Ghiasi et al. [114] developed nitrogen- and sulfur-doped CDs from chicken eggshell membrane for metronidazole aptasensing, demonstrating that eggshell membrane waste can function as a carbon-rich precursor for contaminant detection platforms [114]. However, the final recovery or synthesis yield of the eggshell membrane-derived N,S-CDs was not reported. Shell-derived HAp shows promise for adsorption-based water treatment, while eggshell membrane-derived carbon materials offer potential for sensing and environmental monitoring applications. Future work should address real wastewater validation, adsorbent regeneration, long-term stability, and environmental safety.

For agricultural applications, animal-waste valorization is moving beyond the traditional fertilizer to highly efficient nano-delivery systems. For instance, cow-dung-mediated synthesis of ZnO NPs showed enhanced seed germination, root and shoot growth, and nutrient uptake while exhibiting lower toxicity and reduced reactive oxygen species (ROS) generation compared to commercially synthesized ZnO NPs [115]. Additionally, valorization of waste eggshells, composed of CaCO_3_, provided a sustainable and cost-effective precursor for the synthesis of CaO NPs with multiple applications such as in crop nutrition and protection and antimicrobial activities. Research has demonstrated that optimized thermal decomposition at 900 °C following wet-mill grinding and calcination achieved a CaO NP yield of 97.22%, and when utilized as Ca-rich nano-fertilizer these NPs significantly enhanced seed priming, plant vitality, and soil pH compensation in acidic environments [116]. Furthermore, CaO NPs synthesized via sol-gel methods produced NPs that had an average diameter of approximately 27.7 nm and exhibited potent broad-spectrum antibacterial activity against pathogens such as *E. coli*, *Salmonella enteritidis*, and Methicillin-resistant *S. aureus* (MRSA), with antibacterial performance showing a strong positive correlation with NP concentration [117]. These advances offer solutions to alleviate issues relating to nutrient deficiencies in plants through environmentally safe and sustainable farming.

#### 3.2.3. Energy Systems

In energy applications, animal waste offers a rich source of carbon and metal oxides for high-performance devices. Cow dung extract-derived ZnO NPs have been explored by Suresh et al. [118] in the field of energy conservation as dye-sensitized solar cells. Methanol and ethanol extracts exhibited UV-Vis absorbance between 300–730 nm, indicating the presence of chlorophyll a, chlorophyll b, and carotenoids derived from the herbivorous diet of cows. The presence of methyl groups in chlorophyll facilitated effective bonding with ZnO, enhancing light absorption and electron transport, achieving an energy conversion efficiency of 0.102% under optimized conditions [118]. Beyond solar energy, bovine and porcine bone waste have emerged as precursors for activated porous carbon used in supercapacitors due to their enhanced structural stability, electric and thermal conductivity, high porosity, ease of modification, and flexibility. Due to the natural hierarchical structure of bone, these carbon materials exhibit high surface areas (>2000 m^2^/g) and inherent nitrogen/phosphorus doping, leading to exceptional specific capacitance and long-term cycling stability in energy storage devices [119]. In other studies, carbon nano-onions (CNOs) synthesized from fish scale waste via one-step microwave pyrolysis produced multi-shelled graphene-like cores with visible photoluminescence and a quantum yield of 40%—tenfold greater than previously reported CNOs. This study also reported a pyrolyzed fish scale yield of approximately 51.8% and a solid CNO yield of approximately 29.2% relative to fish scale. This method avoided use of harmful reagents, long reaction time, and costly carbon sources and the nanomaterials were used in solid-state lighting systems and flexible films and LEDs, demonstrating outstanding dispersibility in polar solvents [120]. Such optoelectronic applications highlight the potential of animal waste-derived carbon nanostructures to compete with conventional nanocarbons while offering rapid, reagent-free synthesis routes.

Overall, animal WDNPs represent a highly promising yet underutilized class of sustainable nanomaterials (Table 3). Their unique biochemical composition enables the synthesis of multifunctional nanostructures with applications across biomedicine, environmental remediation, agriculture, and energy systems. Although the benefits are substantial, several challenges remain; these include intrinsic variability in feedstock composition, complex preprocessing requirements, risks of bio-contamination, and challenges in scalability and standardization. Furthermore, regulatory approval and long-term safety evaluation remain significant bottlenecks. Addressing these limitations through integrated process optimization, standardization frameworks, and interdisciplinary collaboration will be essential for translating animal waste-derived nanotechnologies from laboratory research to industrial and clinical applications [89].

### 3.3. Plastic Waste-Derived Nanoparticles

**Figure 4:**
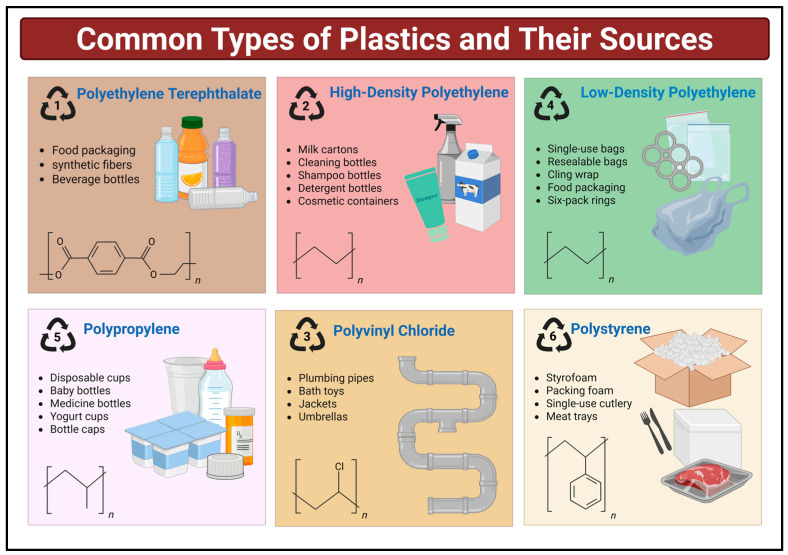
Common types of plastics and their primary sources. Created in BioRender. Yadav, M. (2026) https://BioRender.com/tgoqmkm (accessed on 22 May 2026).

Plastic waste represents one of the most persistent and critical environmental challenges of the modern era, driven by its low production cost, extensive global consumption, and intrinsic resistance to degradation, which collectively lead to extensive land and aquatic pollution. It is estimated that plastic contributes to 60–80% of marine debris and approximately 10% of household waste streams [132]. Over time, environmental, biological, and chemical weathering processes fragment larger plastic debris into microplastics (<5 mm) and nanoplastics (1 nm–1 µm) [133]. This progressive fragmentation not only increases environmental dispersion but also enhances bioavailability and ecological risk due to the increased surface area and reactivity of smaller particles. Beyond physical pollution, plastics represent a substantial source of chemical contamination. Widely used polymers such as polyethylene (PE), polypropylene (PP), polystyrene (PS), polyethylene terephthalate (PET), and poly(vinyl chloride) (PVC) can release hazardous organic compounds including polychlorinated biphenyls (PCBs), polycyclic aromatic hydrocarbons (PAHs) and bisphenol A (BPA) (Figure 4) [132]. PET, despite being the most frequently recycled plastic globally with a recovery rate of approximately 22%, has extensive consumption that contributes to large volumes (millions of tons) that enter the environment annually, exacerbating global plastic pollution [134]. These hydrophobic contaminants readily bioaccumulate and biomagnify through food webs, posing significant ecological and human health risks [135].

Globally, plastic production has reached approximately 400.3 million tons per year and continues to rise. Waste management remains inefficient, with only ~9% recycled and ~12% incinerated, while the majority accumulates in landfills or the natural environment [136]. Projections indicate that cumulative plastic waste could reach 12 billion tons by 2050 if current trends persist. These figures highlight a critical need for advanced valorization strategies capable of converting plastic waste into high-value materials rather than low-value recycled products [137]. Traditional recycling technologies, including mechanical and chemical recycling, often have significant drawbacks. Mechanical recycling degrades polymer chains, reducing material quality, while chemical recycling is energy intensive, costly, and often environmentally burdensome. Furthermore, the heterogeneity and contamination of real-world plastic waste streams significantly reduce process efficiency and product consistency [138]. Consequently, the threat of physical persistence and chemical toxicity of plastic particles and the limitation of traditional recycling techniques have catalyzed interest in alternative upcycling pathways, particularly the conversion of plastic waste into functional NPs, which represent a higher-value and potentially more sustainable end use.

#### 3.3.1. Biosensing, Bioimaging, and Biomedical Systems

Plastic waste can be transformed into functional nanomaterials via thermochemical and physicochemical processes such as pyrolysis, hydrothermal treatment, and solvothermal synthesis [136]. These methods not only mitigate plastic waste accumulation but also generate functional nanomaterials with enhanced physicochemical properties. Carbon- based NPs derived from plastic waste have demonstrated significant potential in biosensing and bioimaging due to their oxygenated surface functional groups (–OH, –COOH) and intrinsic photoluminescence [139]. Hu et al. [140] pioneered the conversion of waste plastic bags into carbon NPs via a hydrogen peroxide-assisted hydrothermal process, yielding particles with high optical properties and high aqueous dispersibility and a maximum CNP yield of 51 wt% under optimized H_2_O_2_-assisted hydrothermal conditions. These NPs exhibited dual functionality, by showing selective and sensitive detection of Fe^3+^ ions, with a detection limit as low as 2.8 μM, and showed strong fluorescence in cellular environments without significant cytotoxicity [140].

Two notable studies further demonstrated the potential of recycling medical plastic waste into luminescent and biocompatible carbon dots (CDs) for bioimaging. In one study, polytetrafluoroethylene (PTFE) syringe waste was carbonized in conjunction with hyaluronic acid to produce CDs with strong fluorescence, high solubility, antibacterial and antifungal activity [141]. These CDs maintained 91% cell viability even at high concentrations (1–2 mg/mL) and enabled high-quality imaging of cells under confocal microscopy. Similarly, intravenous medical bag waste converted into chitosan-functionalized CDs were approximately 2–8 nm in size, highly fluorescent, stable and exhibited potent antibacterial activity against both Gram-positive and Gram-negative bacteria via membrane disruption and oxidative stress [142]. These studies however did not report final recovered CD yield. Although these materials offer excellent optical properties and biocompatibility at low cost, long-term intracellular fate, biodegradation pathways, and regulatory concerns around residual plastic additives remain significant barriers.

#### 3.3.2. Environmental Remediation

In the field of environmental remediation, plastic-derived carbon NPs have demonstrated significant potential due to their low cytotoxicity, high surface area, tunable porosity, and sustainable production [143]. PET is one of the most recycled plastics globally (22% recycling fraction) and its properties and industrial innovations make it a major contributor to environmental pollution. Therefore, studies to convert PET waste to high-value materials are highly needed. El Essawy et al. [144] synthesized graphene NPs from PET via thermal dissociation and applied them to remove harmful dyes from wastewater. The resulting NPs exhibited high surface area and microporosity enabling efficient removal of dyes such as methylene blue and Acid Blue 25. Because of these properties, it was found that the adsorption process took only 30–50 min to reach equilibrium, and the reaction was spontaneous and endothermic with efficiency strongly influenced by temperature and dye concentration [144].

Plastic waste-derived carbon NPs have been investigated for the removal of pharmaceutical contaminants and antibiotic residues from wastewater. Tewari et al. [145] converted single-use waste plastics into reduced graphene oxide sheets and prepared a waste plastic-derived reduced graphene oxide (WrGOs)-Fe_3_O_4_ magnetic nanocomposite for water purification and supercapacitor applications. The composite showed improved removal of diclofenac and caffeine compared with bare WrGOs, with Fe_3_O_4_ functionalization attributed to the enhanced adsorption performance of the plastic-derived graphene material [145]. Miao et al. [146] prepared PP waste-derived N-doped carbon nanotubes (N-pCNTs-5) for sulfamethoxazole degradation in high-salinity wastewater through nonradical peroxymonosulfate activation. The N-pCNTs-5 catalyst achieved complete sulfamethoxazole removal within 30 min, with pyrrolic N identified as the main active site involved in PMS activation and electron-transfer-mediated degradation [146]. These findings indicate that plastic waste-derived carbon NPs can be tailored for pharmaceutical and antibiotic pollutant treatment, particularly under complex wastewater conditions. Similarly, another study focused on the synthesis of porous nanofoams incorporated with Tin (IV) oxide (SnO_2_) NPs from polystyrene plastic waste for the photocatalytic degradation of dye. They used a polymeric precursor method for NPs synthesis and these 20 nm sized NPs embedded into PS foams via thermally induced phase separation, achieved a high surface area of 48 m^2^/g and degraded 98.2% of rhodamine B under UV irradiation. Additionally, they maintained catalytic activity for up to four uses, and enhanced performance was attributed to efficient mass transfer and radical–dye interactions enabled by the porous architecture [147]. Despite these promising remediation performances, product recovery was not consistently reported across these studies. El Essawy et al. [144] and the de Assis study [147] did not provide final material recovery, while Tewari et al. [145] reported 5.75 g of the magnetic nanocomposite from 6 g of the plastic-derived graphene material but did not provide overall yield from plastic waste. In the PP-derived carbon nanotube study, carbon deposition and nanotube purity were reported, but final material yield was not clearly provided in the main text.

#### 3.3.3. Energy Storage and Conversion Systems

The transformation of plastic waste into energy storage materials represents a rapidly growing research area, driven by the demand for sustainable energy technologies. An interesting study of plastic waste reused for energy storage was demonstrated by Li et al. [148], where recycled plastic bottles were transformed into electrospun flexible nanofiber electrodes for supercapacitors. These nanofibers were embedded with acidified multi-walled carbon nanotubes and coated with nano-MnO_2_ film via electrodeposition. These electrodes, when integrated into a symmetric supercapacitor, exhibited excellent performance, with a specific capacitance of 118.8 mF/cm^2^ at a scan rate of 10 mV/s, and maintained high stability with 97.6% capacitance retention even after 5000 charge/discharge cycles. Thus, the multistage porous composite nanofibers exhibited high electrical conductivity and strong pseudocapacitive behavior [148]. In another study, high-performance lithium-ion battery anodes have been developed by embedding SnO_2_ NPs within a hollow carbon sphere/porous carbon flake (HCS/PCF) framework derived from template carbonization of plastic waste. Various morphologies of NPs were synthesized by optimizing the loading content and doping mechanism and among these different shapes, nano-spheres and nano-cubes exhibited the highest electrochemical activity and long-term cycling stability, with an average capacity decay rate of only 0.048% and 0.05% per cycle after 400 cycles and maintained high reversible capacities of 0.45 and 0.498 Ah/g after 1000 cycles. These excellent performances are due to their yolk-shell architecture, which helps in volume expansion of SnO_2_ during lithiation/delithiation processes and in the formation of a stable solid electrolyte layer. This study demonstrated a novel technology of disposing plastic waste to produce materials for more stable lithium-ion battery storage [149].

Energy storage and conversion represent another area where plastic waste-derived NPs have shown utility. Tewari et al. [145] also reported the energy storage application of waste plastic-derived reduced WrGOs-Fe_3_O_4_. When used as a supercapacitor electrode, WrGOs-Fe_3_O_4_ achieved a specific capacitance of 488 F/g at 1 A/g in a three-electrode system. The assembled device delivered an energy density of 52.57 Wh/kg and retained good cyclic stability after 5000 charge–discharge cycles, pointing to the dual utility of plastic-derived graphene-based nanocomposites for both water treatment and energy storage. Taking a different approach, Stevanovic et al. [150] converted PET plastic waste hydrolysates into bacterial nanocellulose (BNC) and used the resulting material as a PVA-reinforced support for Pt NPs. The Pt-BNC/PVA catalyst contained approximately 3 wt% Pt, with Pt NPs around 3.2 nm, and showed methanol oxidation activity for direct methanol fuel cell (DMFC) applications. While this study links PET waste bio-upcycling with electrocatalyst development, the process remains multi-step and dependent on noble metal Pt [150].

Plastic waste has also been used to produce graphene and graphene-like materials using solid-state CVD and molten-salt pyrolysis. Plastic bottles, bags, and containers made of PS, PVC, PP, PET, polymethyl methacrylate (PMMA), and PE are used to produce materials such as graphene foil, and these materials exhibit high electrical conductivity and are suitable for flexible electronics, electro-thermal heaters, and battery electrodes [132,151]. A comparison of these studies also shows that recovery data were reported unevenly. Li et al. reported 95% yield for acidified multi-walled carbon nanotubes, with 23.17 wt% carbon nanotube loading and 35.5 wt% MnO_2_ loading in the final electrode but did not report final plastic waste-derived nanofiber electrode yield. The HCS/PCF-SnO_2_ study reported SnO_2_ loading values of 12.6 to 74.9 wt%, while final HCS/PCF recovery was not provided. In contrast, Stevanovic et al. reported a BNC yield of 3.0 mg/mL under optimized PET-F hydrolysate conditions and 97% yield for Pt-BNC/PVA catalyst synthesis, while the molten-salt graphene study recovered 1.15 g graphitic carbon from 9.83 g PET bottle pieces, corresponding to an approximately 11.7 wt% calculated yield.

#### 3.3.4. Other Emerging Applications

Beyond conventional applications, plastic WDNPs are increasingly being integrated into advanced manufacturing and radiation protection systems. One rapidly emerging area is additive manufacturing (3D printing) where recycled thermoplastics are repurposed into functional filaments, enabling both waste valorization and sustainable manufacturing. A commonly discarded thermoplastic is high-density polyethylene (HDPE) waste, and this has been recycled into 3D printing filaments with high thermal stability, consistent diameters (2.93–3.17 mm), hydrophobicity, and enhanced performance comparable to conventional filaments. Although this study showed successful filament fabrication, it mainly reported filament quality and printability rather than a quantified product recovery or yield. Challenges such as shrinkage and warping existed during printing; these can be mitigated through optimization of extrusion parameters and warping control [152]. Complementary to this work, another study investigated mixed post-consumer plastic waste, mainly PET, PP, and PS that have been compatibilized to overcome the intrinsic immiscibility of polymer mixtures and processed into printable filaments for 3D printing. The incorporation of compatibilizers such as styrene–ethylene–butylene–styrene (SEBS) and SEBS–maleic anhydride improved interfacial adhesion, phase dispersion, and mechanical integrity of the filaments. As a result, recycled PET exhibited the highest tensile strength (35 ± 8 MPa), while PP/PET and PP/PS blends showed moderate mechanical strength and improved ductility. As with the HDPE filament study, the work mainly evaluated processability and mechanical performance rather than final filament recovery. The addition of compatibilizer improved phase dispersion shifted glass transition temperatures, and reduced crystallinity in some formulations, showing that mixed-plastic waste streams can be processed into functional filaments for 3D printing applications. These findings demonstrate that both single-polymer and mixed-plastic waste streams can be effectively processed into filaments for 3D printing applications [153].

In addition to manufacturing applications, plastic waste-derived nanocomposites have also gained attention in radiation shielding, a field traditionally dominated by dense and toxic materials such as Pb. One such novel study of this approach is the use of recycled HDPE as a sustainable matrix to produce lightweight and flexible polymer matrix nanocomposites doped with high atomic number fillers such as CuO NPs and phosphotungstic acid (PTA) to fabricate radiation-shielding nanomaterials. These recycled HDPE/CuO-NP-PTA nanocomposites were synthesized by compression molding using fixed formulation ratios, with 60 wt% recycled HDPE and 40 wt% filler, but the final nanocomposite recovery was not reported. Instead, the study focused on how filler composition influenced structure, thermal stability, and radiation attenuation. The prepared nanocomposites exhibited enhanced gamma-ray mass attenuation coefficient and electron density due to synergistic effects between the polymer matrix and inorganic fillers. Experimental evaluation using gamma sources such as ^133^Ba, ^137^Cs, and ^60^Co confirmed effective shielding performance across a wide energy range, demonstrating the potential of these materials for both stationary and mobile radiation protection applications. Importantly, the lightweight, flexible, and non-toxic nature of recycled HDPE-based NPs offered substantial advantages over conventional shielding materials, especially for protective garments and portable shielding systems in medical, industrial, and nuclear environments [154]. Catalytic pyrolysis also offers a route for converting plastic waste into carbon nanomaterials while simultaneously generating liquid fuel products. Kong et al. [155] investigated the pyrolysis of PP and PS over biomass-derived porous carbon-supported Ni-Fe bimetallic nanocatalysts, resulting in the formation of oil products and CNTs [155]. Although the catalyst was derived from biomass rather than plastic waste, the plastic feedstock served as the carbon source for CNT growth. Under optimized conditions, the 0.2FeNi-MEC catalyst produced approximately 30% CNT yield from PS at 600 °C, with CNT diameters mainly around 40 nm. This study is therefore relevant to plastic waste-derived nanomaterials because it demonstrates direct conversion of plastic carbon into high-value CNTs, rather than limiting plastic upcycling to fuels or small-molecule chemicals.

The upcycling of plastic waste into functional nanomaterials represents a pivotal shift in environmental management, transitioning from polymer pollution management to high-value resource recovery. The primary benefit of this approach lies in the chemical versatility of polymers like PE, PP, and PET, which serve as abundant, carbon-rich precursors for the synthesis of advanced structures like carbon nanotubes, graphene, and porous nanofoams, supporting circular economy principles (Table 4). However, significant technical and economic challenges persist that hinder the widespread industrial implementation of plastic waste to nanomaterials. As with the other waste streams, a major hurdle is the heterogeneity of the plastic waste in which mixed plastics are frequently contaminated with organic residues, dyes, and various additives, which can compromise the purity and properties of the resulting nanomaterials. Furthermore, achieving high-purity separation and thermochemical conversion processes is often energy intensive and requires specialized infrastructure, and the risk of generating secondary nanoplastic pollutants can potentially compromise the overall sustainability of the recycling process. Therefore, future research must focus on developing low-energy, solvent-free synthesis routes and robust mechanical strategies that can accommodate the inherent variability of real-world plastic waste to deliver nanotechnologies that have environmental and societal benefits rather than shifting pollution across life-cycle stages.

### 3.4. Electronic Waste (E-Waste)-Derived Nanoparticles

**Figure 5:**
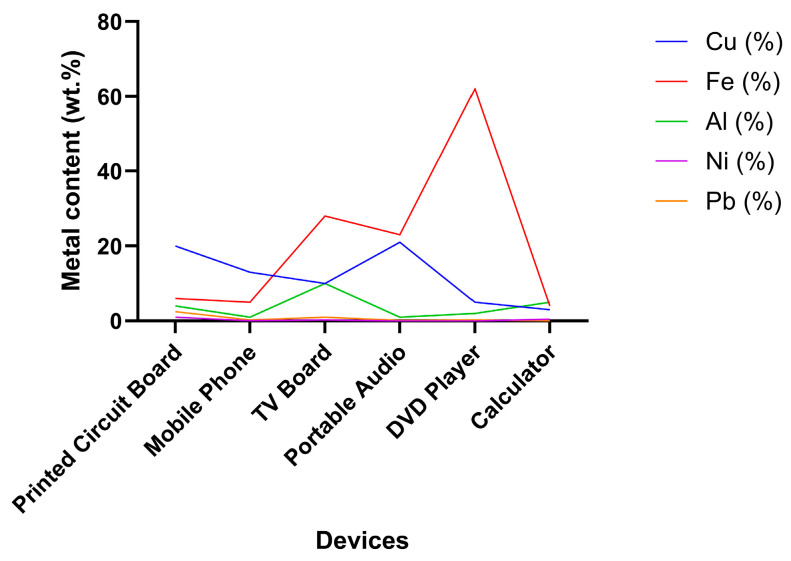
Illustrates the distribution of metals across different e-waste devices, highlighting the significant variability in elemental composition [170].

Globally, a staggering 62 million tons of e-waste are generated annually—a figure projected to increase to 82 million tons by 2030 [166]. Improper management and unregulated disposal of e-waste pose severe environmental and public-health risks due to the release of toxic metals, persistent organic pollutants, and acid-generating components, leading to contamination of soil, water, and ecosystems [167]. The rapid escalation of e-waste production is closely linked to the drastic reduction in product lifespans driven by technological obsolescence and consumer demand. Between 2000 and 2010, the average lifespan of large electronic devices decreased from around 8 years to 2 years, while mobile phones declined from ~4 years to as little as 9 months [168]. This reflects a fundamentally unsustainable consumption model and highlights the urgent need to transition from conventional recycling to high-value material recovery strategies, where waste is not merely processed but functionally upgraded [169]. E-waste includes a wide range of devices, including household appliances, communication equipment, medical devices, and everyday electronics such as televisions, microwaves, batteries, refrigerators and computers. These waste streams contain valuable metals such as Cu, Au, Ag and platinum (Pt), which are recovered using pyrometallurgic routes (Figure 5).

Notably, printed circuit boards (PCBs) represent one of the most metal-dense fractions, containing significant concentrations of rare earth elements including praseodymium, neodymium, lanthanum, and cerium [171]. However, the intrinsic heterogeneity of e-waste, comprising metals, polymers, ceramics, and glass, renders conventional recycling both technically challenging and economically inefficient. Mechanical separation methods are increasingly ineffective due to device miniaturization and material integration, particularly in low-resource settings [172]. Consequently, transformation of recovered materials into functional NPs represents a paradigm shift from resource recovery to value-added nanomaterials, enabling circular utilization of complex waste streams. However, this transition is not without trade-offs; while e-waste offers high-value precursors, the recovery processes are often energy-intensive and chemically demanding, raising critical concerns regarding sustainability when evaluated across the full life cycle [173].

#### 3.4.1. Biomedical Systems

E-waste-derived NPs show strong potential in biomedicine due to their unique surface chemistry and redox activity. For instance, CuNPs recovered from waste PCBs using bioleaching and subsequently chemical reduction generated fine Cu crystallite particles (27.6 nm in size), comprising both metallic Cu^0^ and CuO phases. Notably, the dual oxidation states (Cu^0^/Cu^2+^) enhance redox activity, enabling applications in antimicrobial therapy, biosensing, and drug delivery. However, this same reactivity introduces a critical limitation, increased cytotoxicity and oxidative stress, necessitating rigorous dose-dependent and long-term biocompatibility assessments before clinical translation [174]. Similarly, in a landmark advancement for green electrochemistry, Moriwaki et al. [175] reported a novel electrochemical strategy for extracting AuNPs directly from e-waste using alternating current (AC) in the presence of phospholipid stabilizers 1,2-dioleoyl-Sn-glycero-3-phosphocholine (DOPC). This single-step electrochemical strategy bypasses the requirement for corrosive reagents such as aqua regia or cyanide, typical of conventional Au recovery, but instead used e-waste as an electrode immersed in a 4-(2-hydroxyethyl)-1-piperazineethanesulfonic acid DOPC solution. The resulting phospholipid-coated AuNPs exhibited enhanced colloidal stability and biocompatibility, making them suitable for drug delivery and photothermal therapy. Importantly, this approach illustrates how process design can simultaneously address sustainability and functionality—a key requirement for next-generation nanomaterial synthesis [175].

A further remarkable example involves the synthesis of Mn/ZnO-nanocomposites from spent batteries combined with *Borassus flabellifer* (toddy palm) plant extracts. These NPs (average size of 20 nm) demonstrated significant antimicrobial activity against *S. aureus*, *E. coli*, and *P. aeruginosa*, as well as significant anticancer activity across multiple cell lines, including MDA-MB-231 (breast), SKOV-3 and OVCAR-3 (ovarian), and BxPC-3 (pancreatic) [176]. Such hybrid approaches illustrate the potential for integrating green chemistry with waste valorization.

E-waste-derived NPs have moved beyond simple metal recovery toward multifunctional antibacterial and anticancer platforms. Bharti et al. [177] synthesized Ag-MnO_2_/PIn nanocomposites using MnO_2_ recovered from discarded batteries and Ag NPs prepared using sugarcane husk extract [177]. The resulting nanocomposites showed antibacterial activity against *Pseudomonas aeruginosa* and *Staphylococcus aureus*, with inhibition zones of up to 21.5 mm and 21 mm, respectively. Concentration-dependent cytotoxicity was also reported against pancreatic cancer cell (BXPC-3), ovarian cancer cell (OVCAR-3), triple-negative breast cancer cell (MDA-MB-231), and ovarian cancer cell (SKOV-3) cell lines, with 88.5 to 93.7% inhibition at 3000 nM. While this study effectively combines battery waste valorization, green synthesis, and biomedical testing, normal-cell toxicity assessment, mechanistic studies, and in vivo validation remain necessary before clinical translation.

A common limitation across these biomedical examples is the limited reporting of final NP yield. The PCB-derived CuNP study reported Cu concentrations after bioleaching but did not quantify final CuNP yield, while the Mn/ZnO-NC and Ag-MnO_2_/PIn studies described synthesis from discarded batteries without reporting final nanocomposite recovery. In contrast, Moriwaki et al. [175] provided clearer recovery data, reporting 35% Au extraction from gold-coated sensor-chip components and 214 µg Au recovered at 100 V in the gold-wire model system.

#### 3.4.2. Advanced Photocatalytic and Energy Systems

Photocatalysis has emerged as a cornerstone of environmental remediation due to its ability to mineralize organic contaminants and neutralize microbial pathogens through the generation of ROS, such as hydroxyl radicals and superoxide anions. An ideal photocatalyst must be cost effective, chemically stable, non-toxic, and exhibit high quantum efficiency under broad light spectra. Metallic and semiconductor NPs, such as TiO_2_ and ZnO NPs, remain benchmark materials in photocatalysis and their recovery from e-waste provides a significant sustainability advantage [178].

Niu et al. demonstrated the potential of discarded multilayer ceramic capacitors (MLCCs) as precursors for high-performance photocatalysts [179,180]. In one study, Niobium–palladium (Nb-Pd) co-doped and Pd-loaded TiO_2_/BaTiO_3_ heterostructures were fabricated via chlorination–leaching process, yielding particles of 20–50 nm in size. These materials exhibited enhanced charge separation, improved visible light absorption, and narrowed bandgaps (2.81–2.92 eV), resulting in photocatalytic performance up to 19-fold higher than commercial TiO_2_ [180]. In a subsequent study, discarded MLCCs were reused to fabricate Nb-Pd co-doped BaTiO_3_/Ni-Pd@g-C_3_N_4_ core-shell Z-scheme photocatalysts through ball milling, achieving H_2_ evolution and RhB degradation activities 22.2 and 19.1 times higher than g-C_3_N_4_, respectively. This study also reported product recovery, obtaining 4.96, 5.04 and 5.12 g of photocatalyst from 5 g of waste-capacitor-derived powder with 5, 10 and 20 wt% g-C_3_N_4_, respectively. These improvements were attributed to engineered heterojunctions, emphasizing that functional performance is governed more by material design than by precursor origin alone [179].

Beyond capacitors, spent batteries represent an abundant secondary source of Zn. With the increased usage of portable electronic devices, the global demand for Zn-C and Zn-Mn batteries has increased significantly, making them an important source of Zn. ZnO nanosheets successfully synthesized from spent Zn-C batteries through a simple precipitation-calcination technique formed zinc hydroxide nitrate hydrate (Zn_5_(OH)_8_(NO_3_)_2_(H_2_O)_2_) porous nanosheets with thicknesses up to 100 nm and efficient methylene blue degradation kinetics [181]. Similarly, Zhan et al. [182] used high-temperature vacuum evaporation methods yielding tetrapod-shaped ZnO NPs from spent Zn-Mn batteries. Under ideal circumstances, high-purity NPs were fabricated with remarkable UV absorption and photocatalytic capability, which are critical for high-performance photocatalytic and optoelectronic devices [182].

Battery-derived carbon NPs have been coupled with molybdenum disulfide (MoS_2_) and tungsten disulfide (WS_2_) to form CNPs@MoS_2_ and CNPs@WS_2_ composites for hydrogen evolution reactions, showing how spent battery carbon can be reused to improve conductivity and catalytic activity in energy storage and electrocatalysis. Spent lithium ferromanganese phosphate battery cathodes and anode graphite have similarly been converted into MnFe_2_O_4_-reduced graphene oxide (rGO) composites for Li-CO_2_ battery cathodes. These examples indicate that e-waste-derived NPs are not limited to pollutant degradation but can also support sustainable energy conversion and storage [183,184]. Some hybrid systems, however, still rely on commercial co-catalysts or complex chemical pretreatment, which should be factored in when evaluating their overall sustainability.

#### 3.4.3. Environmental Remediation Systems

PCBs represent one of the most metal-rich fractions of e-waste, with Cu as a dominant component. Several studies have investigated the role of PCB-derived CuO NPs for environmental remediation, particularly in the degradation of dyes such as MB, Congo red, and methyl orange. Gautam et al. [185] synthesized monoclinic CuO NPs via alkaline precipitation, generating uneven polycrystalline particles with a band gap of 1.93 eV, achieving 98.02% degradation of methylene blue under visible light irradiation. The narrow bandgap and visible-light activity represent a significant advantage over conventional TiO_2_ photocatalysts, which require UV activation [185]. Complementary studies utilized *Ocimum sanctum* leaf extract as a reducing agent to produce spherical CuO NPs (14.6–19.7 nm), achieving 68.4% degradation of methyl orange after 24 h [186]. These studies collectively demonstrate that structural engineering, including porosity and surface functionalization, is a key determinant of catalytic performance, suggesting future research should prioritize morphology-controlled synthesis rather than solely focusing on metal recovery.

Hossain and Ahmed synthesized nano-CuO directly from waste electric cable using acid dissolution and alkaline precipitation. The product was mainly tenorite CuO, with crystallite sizes ranging from 7 to 14 nm, and Rietveld refinement confirmed 99.524% CuO with only 0.476% Cu_2_O [187]. While this study focused primarily on structural characterization rather than direct remediation testing, it provides a clear example of converting Cu-rich e-waste into high-purity metal oxide NPs. Nithya et al. [188] took a different approach, developing a circular bioeconomy strategy using Cu-containing e-waste from PCBs and *Prosopis juliflora* biomass to synthesize green CuO NPs. The resulting gCuO NPs had crystallite sizes of 15 to 25 nm and achieved more than 90% removal of pharmaceutical contaminants including diclofenac, bisphenol-A, and carbamazepine [188]. This study integrates e-waste valorization, plant-mediated synthesis, response surface methodology (RSM) and artificial neural network (ANN)-based optimization, regeneration over multiple cycles, and life-cycle assessment (LCA). The reported reductions in global warming potential and cumulative energy demand compared with conventional CuO synthesis position it as a strong example of sustainable e-waste-derived nanomaterials for wastewater treatment.

The valorization of e-waste into functional nanomaterials represents a transformative step toward a circular “waste-to-wealth” economy, offering both ecological and economic benefits. E-waste serves as a rich, low cost secondary reservoir of transition metals and semiconductors that can match or exceed the performance of commercially sourced materials for application ranging from advanced photocatalysts to biomedical systems. However, despite these promising breakthroughs, several hurdles remain that hinder the large-scale industrial adoption of electronic WDNPs. The most significant challenge lies in the heterogeneity and structural complexity of modern electronics, where devices are increasingly composed of intricate circuit systems, and where high-purity separation remains technically demanding and energy intensive. Furthermore, the recovery processes, which often involve the use of concentrated acids or high temperature calcination, must be strictly regulated to prevent secondary environmental pollution, which could potentially offset the green benefits of the recycling effort. Therefore, future research must move beyond isolated recovery or synthesis approaches and instead focus on integrated, low-energy recovery–synthesis systems, to ensure that the transition from hazardous e-waste to high-value nanomaterials is both economically viable and ecologically sustainable on a global scale.

#### 3.4.4. E-Waste-Derived Materials for Metal Recovery and Catalysis

E-waste-derived systems have also been applied to selective metal recovery and catalytic upcycling, shifting recycling strategies from simple recovery toward high-value circular material production. Zadehnazari et al. [189] recovered Au from waste CPU-derived metal flakes using tetrazine-linked covalent organic frameworks (COFs). Tetrathiafulvalene (TTF)–COF showed an Au(III) adsorption capacity of 2440 mg g^−1^ and selectively captured >99% of Au from CPU-derived leachate, while TPE-COF achieved 99.11% Au recovery. The recovered Au was captured and reduced within the COF structure, forming Au-loaded COF catalysts containing mainly spherical Au NPs of approximately 5 to 10 nm. These catalysts were applied for terminal alkyne carboxylation using CO_2_, demonstrating a dual valorization strategy in which e-waste-derived Au was converted into a reusable catalyst for green organic transformation. However, the COF framework itself was synthetically prepared; therefore, only the Au component can be considered e-waste-derived [189].

Other studies have focused on sustainable Au recovery rather than direct NP synthesis. Mann et al. [190] developed a cyanide-free and mercury-free approach for gold extraction from ore and electronic waste using trichloroisocyanuric acid and NaBr for leaching, followed by selective gold capture using polysulfide polymer sorbents. When applied to RAM-derived e-waste concentrate, >97% of Au was bound by the polymer sorbent, producing a final refined gold bead of 3.34 g with 99.9% purity and 86% isolated yield. Copper was also recovered from the remaining leachate in >98% yield by electroplating [190]. Chen et al. [191] developed a Cu NP-modified electrochemical Ni membrane reactor for Au recovery from acidic CPU-derived leachate. This system achieved complete Au recovery from 20 mg/L AuCl_4_^−^ solution with a unit energy consumption of 0.13 kWh gAu^−1^, and also demonstrated 100% Au recovery from real CPU-derived leachate with separated Au purity above 99 atomic% [191]. While these studies fall outside direct NP synthesis from e-waste, they illustrate how metal recovery from e-waste can feed into the production of value-added catalytic and functional materials within a circular economy framework. Table 5 represents the recent studies utilizing e-waste to generate NPs.

### 3.5. Industrial Waste-Derived Nanoparticles

The term “industrial waste” refers to discard materials generated from industrial processes like mining, manufacturing, milling and chemical production. Broadly, industrial waste can be categorized into two types: biodegradable and non-biodegradable streams, each presenting distinct management challenges [203]. Biodegradable industrial wastes consist of materials that can be easily decomposed through microbial activity without generating highly toxic by-products. These include waste streams from food processing, textiles, paper production, and dairy industries, which generate materials such as leather residues and paper sludge. While these materials are generally less hazardous and easier to manage, their large volumes and high organic load can still contribute to environmental issues such as eutrophication if improperly managed [203]. In contrast, non-biodegradable industrial waste cannot be readily broken down into non-toxic components and therefore poses longer-term environmental risks. These wastes originate from industries such as steel manufacturing, chemical processing, fertilizer production, pharmaceuticals, and dye industries, generating residues including fly ash, slag, and complex chemical by-products. Currently, non-biodegradable waste accounts for approximately 15% of the global industrial waste burden, a figure that continues to rise with accelerating industrialization. Traditional management strategies rely heavily on energy-intensive incineration or landfilling, although some residues such as steel slag and fly ash are repurposed for cement manufacturing. While such reuse reduces landfill burden, it often represents downcycling rather than high-value material recovery [204].

A paradigm shift is therefore emerging, where industrial waste is increasingly recognized as a secondary resource for high-value NP synthesis, aligning with circular economy principles. Pretreatment represents a critical step in this process, as both the treatment method and inherent chemical composition of the waste directly influence NP physicochemical properties, including size, surface chemistry, and reactivity. Techniques such as thermal activation, acid leaching, and mechanical homogenization are commonly employed; however, the absence of standardized pretreatment protocols remains a major barrier to reproducibility and scalability. This variability introduces significant uncertainty in NP performance, particularly for applications requiring high precision such as catalysis and biomedicine. Consequently, standardization of preprocessing frameworks is essential for translating laboratory-scale success into industrial implementation [205].

Industrial waste streams are particularly attractive due to their high content of valuable elements such as Cr, Cu, Fe, Ni, Ti, and Al. These materials can be converted into metal, metal oxide, and carbon-based NPs via thermochemical and physicochemical processes including coprecipitation, pyrolysis, calcination and ball milling. While these approaches enable effective resource recovery, they often involve energy-intensive processing steps, raising important questions regarding their overall sustainability when assessed from a life-cycle perspective [203,205].

#### 3.5.1. Environmental Remediation Systems

Industrial waste NPs have demonstrated significant potential in environmental remediation due to their high surface area, tunable surface chemistry, and strong adsorption capabilities. Fly ash, a fine granular solid by-product of coal combustion, is rich in silicon dioxide (SiO_2_), Al_2_O_3_, and CaO, making it an ideal precursor for silica-based NPs [206]. These SiNPs are widely applied in wastewater treatment for the removal of heavy metals and organic pollutants, often achieving removal efficiencies exceeding 90%. From a material recovery perspective, fly ash-derived biosilica provides one of the clearer quantitative examples, with Liang et al. [207] reporting a biosilica yield of 20.45% and an increase in SiO_2_ purity from 44.41% in raw fly ash to 93.63% in the final product. Their performance is largely attributed to abundant surface functional groups that facilitate adsorption, while integration into hybrid photocatalytic–adsorptive systems further enhances degradation efficiency [207]. Wang et al. [208] presented an innovative application of fly ash NPs in stabilizing CO_2_ foams for the remediation of non-aqueous phase liquid (NAPL) contamination. By combining fly ash NPs with surfactants (AOS and LAPB), a highly stable foam system was developed that achieved ~45% removal of residual fuel oil in porous media models. While this represents a significant improvement over conventional waterflooding techniques, a critical limitation was identified: excessive NP loading led to pore blockage and reduced permeability, highlighting the existence of an operational concentration threshold [208].

Red mud, a highly alkaline solid by-product generated during the extraction of alumina from bauxite ore, represents a highly promising yet underutilized resource. With iron content ranging from 45–60%, alongside Al, Ca, Si, and Ti oxides, red mud exhibits strong catalytic potential for advanced oxidation processes [209]. Due to its high iron content and surface reactivity, red mud has attracted attention for environmental remediation, particularly for the removal of toxic contaminants from water systems. Several studies have shown the use of red mud to synthesize NPs for the removal of arsenic (As) from wastewater where particles ranging from 200 nm to 20 nm and increased As(V) removal efficiency from 58% to over 82% due to enhanced surface area and exposure of active adsorption sites [210]. More advanced nanostructuring approaches have further improved performance. For instance, nanogel-encapsulated red mud systems achieved 99.95% arsenic immobilization efficiency, even under extreme pH conditions [211], while electrospun nanofibres (58 nm diameter) exhibited a maximum As(V) adsorption capacity of 147.71 mg/g and maintained high performance over five regeneration cycles, and strong monolayer chemisorption with strong affinity and reusability [212].

Iron-rich industrial solid wastes have been converted into magnetic adsorbents, photocatalysts, sonocatalysts, and advanced oxidation catalysts for water remediation. Red mud from the Bayer alumina process, for instance, has been used to prepare Fe_3_O_4_/chitosan nanocomposites through ultrasonic-assisted co-precipitation, where the chitosan coating improved surface functionality and produced a magnetic adsorbent with a hydrodynamic size of around 165 nm, surface area of 93.64 m^2^/g, and 96.73% As(III) removal at pH 4 [213]. Despite these results, acid treatment, alkalinity management, and validation in real wastewater remain challenges that need to be addressed before practical application. These studies illustrate the versatility of red mud as a valuable resource for NP and nanostructured material synthesis; nevertheless, large-scale application remains constrained by alkalinity management, potential heavy metal leaching, and lack of life cycle assessments, which must be addressed to ensure environmental safety.

Beyond iron-rich wastes, a broader range of metal-containing industrial residues have been converted into functional nanocatalysts for dye and antibiotic-contaminated wastewater treatment. Aluminium-containing pharmaceutical packaging waste has been used as a precursor for algae-assisted Al_2_O_3_ NPs [214], while iron ore tailing and Raney nickel catalyst processing waste have been combined to synthesize magnetic NiFe_2_O_4_ NPs [215]. Although these studies used different waste sources and synthesis routes, both follow the same circular economy concept, where industrial residues are upgraded into water treatment materials. The Al_2_O_3_ NPs showed rapid Congo red adsorption, achieving 97.81% removal within 30 min, whereas the NiFe_2_O_4_ NPs showed visible-light- and ultrasound-assisted degradation of Reactive Red 35 dye, antibacterial activity against *E. coli*, and reusability over five cycles. Paint sludge-derived cobalt NP-embedded activated carbon further extends this approach into advanced oxidation systems. The resulting Co-AC catalyst contained 5.87 wt% Co and activated peroxymonosulfate efficiently, removing 97% tetracycline within 5 min [214]. These findings indicate that Al-, Fe-, Ni-, and Co-containing industrial waste can be transformed into adsorptive, catalytic, and antimicrobial nanomaterials for wastewater remediation. Practical application, however, remains constrained by chemical pretreatment, calcination or pyrolysis requirements, possible metal leaching, oxidant demand, and limited testing under real industrial wastewater conditions.

Spent catalysts represent a carbon-rich industrial residue that can be processed into high-value adsorbents. Spent Mo/HZSM-5 catalyst-derived CNTs have been incorporated with graphene oxide (GO) to form a three-dimensional GO/CNT hybrid aerogel for phenol adsorption. Rather than functioning as a simple waste-derived powder, this material uses the structural advantages of liberated CNTs and GO to create a porous network with improved adsorption capacity. The hybrid aerogel achieved a Langmuir adsorption capacity of 204 mg/g, pointing to the potential of spent catalyst waste as a source of advanced carbon nanostructures. The need for hydrofluoric acid (HF) during CNT liberation and further regeneration validation, however, remain key concerns before practical application [216].

#### 3.5.2. Biomedical and Biological Applications

The biomedical sector has increasingly explored industrial WDNPs for drug delivery and therapeutic applications due to their cost-effectiveness, sustainability, biocompatibility and multifunctional properties. For instance, lignin, a major by-product of the paper and pulp industry, has emerged as a valuable biopolymer for NP synthesis, particularly as nanocarriers. Lignin-derived NPs exhibit biocompatibility, intrinsic antioxidant activity and tunable surface chemistry, enabling efficient drug encapsulation, targeted delivery and controlled drug release [217]. Utilizing high-pressure homogenization and hydrotropic techniques, researchers have synthesized lignin-based polymeric NPs conjugated with folic acid and polyethylene glycol (PEG), yielding uniform particles (~150 nm), and demonstrated excellent biocompatibility, high drug-loading capacity (~24.2 wt% for hydroxyl camptothecin), extended systemic circulation and a 5-fold increase in cellular uptake. In vivo xenograft models confirmed improved tumor suppression and reduced systemic toxicity due to the folic acid-mediated targeting and PEG-induced stealth effect, confirmed by biodistribution analysis. In vivo body weight of treated mice showed no noticeable change throughout the study. IgE levels in NP-treated groups showed only slight changes compared with the PBS, whereas free HCPT caused higher IgE levels, indicating reduced hypersensitivity reactions. In vivo hematological analysis showed that white blood cell and platelet counts in NP-treated groups remained within an acceptable range, while free HCPT caused marked reductions in both parameters, with no severe haematotoxicity observed [218].

Another valuable industrial waste stream is the residual meal generated by commercial oilseed processing facilities, where large-scale extraction of rapeseed, soybean and sunflower oils produces substantial quantities of protein and fiber-rich solid by-products. Das Purkayastha et al. [219] synthesized multifunctional CNPs from rapeseed oilcake via hydrothermal carbonization, obtaining approximately 171 mg of CNPs per g of dry meal [219]. The resulting CNPs exhibited high hemocompatibility, and antioxidative and potent antimicrobial activity, including disruption of bacterial membranes and plasmid condensation. Upon acetone precipitation, these NPs self-assembled into micrometer-sized spheres with a reported microsphere yield of 87 ± 6%. These microspheres showed a protein loading capacity of 68 ± 6% and aided in the encapsulation and thermal protection of sensitive molecules such as proteins and peptides without major structural disruption, offering a sustainable platform for biomedical, food preservation and environmental monitoring.

#### 3.5.3. Other Novel Applications

Waste tires represent a significant global environmental challenge due to their non-biodegradable nature, complex composition and high annual generation. Tires are composed of approximately 45–47% natural rubber, 21–22% carbon black, 12–25% metals, 5–10% textile fibers, 6–7% additives, 1–2% Zn, and about 1% sulfur (S) [220]. Since tires primarily contain 81% of carbon by weight, they are particularly attractive precursors for the synthesis of carbon-based nanomaterials [3]. Maroufi et al. [221] synthesized carbon NPs using high-temperature pyrolysis (1550 °C), which were spherical (30–40 nm) and exhibited a high surface area (117.7 m^2^/g) suitable for applications in energy storage and sensing [221]. Similarly, Gómez-Hernández et al. [222] reported a simple one-step thermal transformation method for producing carbon black NPs (~22 nm) from waste tires, obtaining 81 g of carbon black NPs from 100 g of tire-derived rubber, corresponding to an approximate yield of 81% [222]. Applications included energy storage, sensing, catalysis, pigments, and concrete modification. However, controlling oxidation levels remains a key challenge, as surface chemistry strongly influences electrical and catalytic performance. Beyond traditional thermal routes, microwave-assisted pyrolysis has emerged as a more energy-efficient alternative, enabling the synthesis of bamboo-shaped carbon nanofibers (425 to 881 nm) from waste tire-derived char under optimized conditions of 1000 W, 40 min irradiation and 100 wt% catalyst loading [223]. Although char yield was considered during precursor preparation, the final CNF yield was not quantitatively reported, and the authors noted that increasing microwave power reduced CNM yield. While these materials show significant advances, the reliance on catalysts and high-energy input still raises concerns regarding scalability and environmental impact.

Mining residues have received comparatively less attention than other industrial waste streams, yet coal tailings have recently been converted into silica/carbon-based nanocomposites using high-energy planetary ball milling. Under optimized conditions of 5 mm balls, a 30:1 ball-to-powder ratio, and 16 h milling, the resulting materials showed particle sizes of 30 to 60 nm. The nanocomposites displayed mesoporosity, mixed hydrophilic and hydrophobic surface groups, weak magnetic behavior, and high thermal stability, suggesting potential applications in adsorption, enhanced oil recovery, carbon capture-related systems, and industrial remediation [224]. Unlike application-focused studies, this work primarily provides synthesis and characterization data, and future studies should validate pollutant removal performance, stability, and behavior in real systems.

Industrial WDNPs represent a powerful strategy for transforming high-volume, non-biodegradable residues into value-added functional nanomaterials, supporting both environmental remediation and resource recovery. The key advantage lies in the abundance and compositional diversity of industrial waste, enabling the synthesis of a wide range of nanomaterials with tunable properties (Table 6). However, this area faces significant technical and logistical hurdles that complicate large-scale implementation. The inherent heterogeneity of industrial waste remains a critical challenge, due to changes in the chemical composition of slag, ash, or ore tailings that can lead to inconsistencies in the structural and functional properties of the resulting NPs. Furthermore, the synthesis process, which often involves high-temperature and energy intensive methods, poses risks of secondary environmental pollution and high operational costs. Additionally, potential leaching of toxic trace elements and the lack of regulatory frameworks for waste-derived nanomaterials remain critical barriers to large-scale adoption. Future work must focus on the development of standardized, automated preprocessing technologies that can ensure the high-purity output required for advanced nanotechnology. It must prioritize low-energy, solvent-free synthesis routes supported by life-cycle and techno-economic assessments. Without these advances, industrial waste-derived nanotechnology risks remaining a promising concept rather than a viable industrial solution.

## 4. Scale-Up and Translational Status of Waste-Derived Nanoparticles

Trial-stage and pre-commercial translation of WDNPs remains limited, where most reported systems remain at Technology Readiness Levels (TRLs) 2–5, in which proof-of-concept performance has been demonstrated but pilot-scale validation remains limited. The clearest examples come from waste-derived carbon nanomaterials, particularly CNTs and graphene, rather than conventional metal or metal oxide NPs. For plastic waste, Nanomatics, a spin-off from Nanyang Technological University in Singapore, has developed a modular pyrolysis process converting plastic waste into CNTs, pyrolysis oil, and hydrogen-rich gas [232]. According to company reporting, each module processes 600 kg/day of plastic waste and produces 80 kg/day of CNTs, 240 kg/day of pyrolysis oil, and 60 kg/day of hydrogen-rich gas, with the process described as TRL 7. These figures have not appeared in peer-reviewed literature and should be read as company-reported only. Universal Matter operates a Flash Joule Heating (FJH) platform that accepts a range of feedstocks including biomass, recycled plastic and rubber, food waste, tire waste, and mixed plastic waste, with lab/pilot graphene production of up to 20 kg/day at its Houston center [233]. The technical feasibility of converting end-of-life vehicle plastic waste into flash graphene via FJH has been independently demonstrated by Wyss et al. [156] in a peer-reviewed study published in Communications Engineering, which also conducted a prospective life cycle assessment showing substantial reductions in cumulative energy demand, global warming potential, and water use compared to conventional graphene production methods [156]. Both cases should be read as company-reported commercialization pathways rather than independently verified large-scale production systems.

Waste gas and biogas-derived carbon NPs have progressed further toward scale-up than most other waste-derived NP categories. United Utilities secured £9.5 million in Ofwat funding to scale Levidian’s LOOP technology, which splits wastewater-derived biogas methane into hydrogen and graphene, following an earlier demonstration at Davyhulme wastewater treatment works in Manchester, with the scaled unit expected to produce roughly three times the output of the earlier system [234,235]. Carbon Corp received $10.3 million from Emissions Reduction Alberta and a separate $3.5 million from the Canadian federal government to convert CO_2_ into CNTs, with a pilot plant operating in Calgary and Capital Power planning a commercial-scale CNT facility at its Genesee power station targeting 2500 tons of CNTs per year. However, for most other WDNP categories, evidence for pilot or pre-commercial scale production remains limited, with the majority of systems reported only at laboratory or proof-of-concept stage. Understanding why most WDNP systems remain at laboratory stage requires closer examination of the synthesis, standardization and safety challenges discussed below.

## 5. Challenges in Waste-Derived Nanomaterial Synthesis Technologies

**Figure 6:**
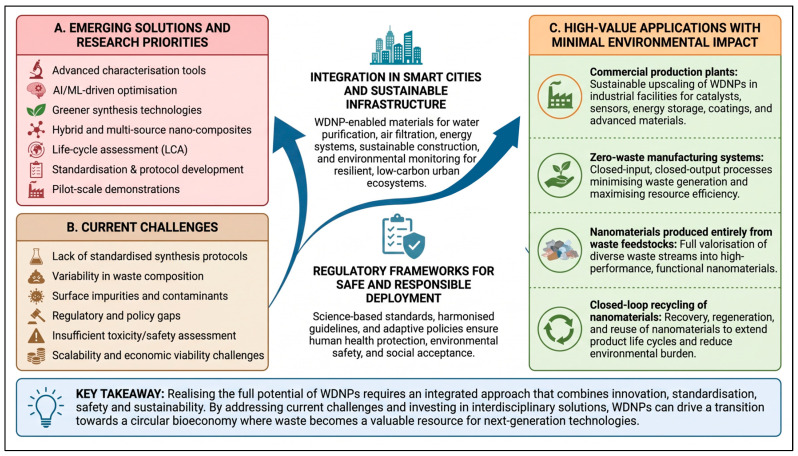
Roadmap for advancing waste-derived NPs. Transition of WDNPs from laboratory-scale demonstration to industrial and societal impact requires coordinated advances across multiple sectors. Created in BioRender. Yadav, M. (2026) https://BioRender.com/mqc8b73. (accessed on 20 May 2026).

The valorization of diverse waste streams, including electronic, agricultural, plastic, industrial and animal-derived waste into NPs, represents a promising pathway toward sustainable material engineering. However, despite strong conceptual alignment with circular economy principles, these advantages remain highly context-dependent and are not yet readily translatable to industrial-scale deployment. As highlighted in the literature, the transition from laboratory innovation to real-world implementation is constrained by a series of interconnected technical, environmental, and regulatory challenges. These include poor reproducibility, complex purification requirements, potential toxicity, long-term environmental uncertainties, high production costs, instability, batch-to-batch variability, and the absence of standardized synthesis and validation protocols. Critically, these limitations are not isolated but mutually reinforcing; for example, variability in waste composition directly impacts reproducibility, which in turn complicates standardization and regulatory approval. As such, overcoming these barriers requires a systems-level approach rather than isolated technical optimizations. These challenges are summarized in the roadmap presented in Figure 6.

### 5.1. Potential Toxicity and Environmental Impact

Despite the expanding applications, the environmental and biological safety of WDNPs remains insufficiently understood. While these materials are often positioned as “green” alternatives, their synthesis is frequently energy-intensive and may inadvertently generate secondary waste streams with unintended ecological consequences. Importantly, the nature and severity of these risks are strongly dependent on the originating waste feedstock [236]. For biologically derived systems (e.g., agricultural and animal waste), a primary concern is inherent variability and environmental instability of the biomolecular or phytochemical capping layer, commonly referred to as the “green corona”. This surface layer, while initially beneficial for stabilization, is highly dynamic and susceptible to degradation, oxidation, or replacement by environmental biomolecules. Interactions with natural organic matter and environmental components alter NP surface chemistry, aggregation, and stability under realistic conditions, directly affecting their environmental fate and biological interactions. This corona evolution is particularly critical for materials applied directly in agroecosystems, as it can expose the reactive core and thereby alter the dissolution rates, bioavailability, and long-term ecotoxicity [237]. In contrast, plastic, electronic, and industrial WDNPs present a different class of risk associated with the co-extraction or retention of hazardous substances. Toxic elements such as Pb, Cd and brominated flame retardants, mobilized during leaching, thermal treatment and other recovery processes, persist through NP synthesis processes or are released as fine particulate by-products or gaseous emission. This raises a critical issue in the field where materials intended for environmental remediation may themselves introduce secondary contamination pathways if not rigorously controlled [238].

Another fundamental challenge resides in understanding NP transport, transformation and biological interactions under realistic environmental conditions. Detection and quantification of trace levels of NPs in complex environmental matrices require more advanced and highly sensitive analytical techniques that are currently still under development [239]. Another crucial issue is that most toxicological data still come from simplified systems, despite WDNP exposure scenarios being multicomponent and chronic. The US Environmental Protection Agency (USEPA) acknowledges that NPs have unique physicochemical properties that make their toxicological assessment challenging. Their small size allows them to cross biological membranes and accumulate in tissues and organs, while high surface reactivity and aggregation behavior in liquids complicate analysis and characterization [240]. Environmental transformations like oxidation, UV exposure and ROS interaction can lead to unintended surface modifications. These changes affect how substances behave in the environment, their transport and ultimately their toxicity profiles [241]. Long-term and multigenerational studies underscore these concerns. For example, research on cerium oxide nanoparticles revealed that acute toxicity tests alone are not enough to assess long-term effects. Sub-chronic and chronic toxicity assessments are therefore necessary [242]. Similarly, Yen et al. [243] used *C. elegans* to demonstrate that long-term exposure to TiO_2_ nanoparticles significantly reduced reproduction survival and growth across successive generations. Furthermore, toxicity effects became more pronounced in later cohorts. Collectively, these findings highlight that safety assessment must evolve from short-term validation to predictive, systems-level evaluation of environmental fate and impact.

### 5.2. Reproducibility and Batch-to-Batch Variability

A recurring concern in WDNP synthesis is poor reproducibility, primarily due to the inherent heterogeneity and compositional variability of waste streams. Unlike conventional methods, which provide more control over reactants and conditions, green and waste-based synthesis often depends on complex mixture of natural or secondary compounds whose composition can vary significantly [238]. For instance, in agricultural and plant-mediated systems, factors such as geographical locations, species variation, seasonal fluctuations, maturity stage and post-harvest handling influence biochemical composition, leading to variability in NP formation efficiency and properties. These variations directly affect reduction kinetics, nucleation behavior, and stabilization mechanisms, resulting in inconsistent particle size, morphology, and functional performance. Similarly, e-waste and mixed industrial waste streams exhibit variability in metal composition, polymer fractions, and contaminant profiles, leading to unpredictable synthesis outcomes. Such variability is incompatible with the stringent quality requirements of high-value applications in catalysis, electronics, biomedicine, and environmental remediation [33,173]. Critically, this challenge is not merely technical but structural; without standardized feedstock characterization and preprocessing protocols, reproducibility will remain an intrinsic limitation.

### 5.3. Purification and Product Consistency

Purification remains a formidable bottleneck that is often underestimated in laboratory-scale studies and becomes problematic at a large scale. Waste materials often yield a complex mixture of proteins, polyphenols, lipids, carbohydrates, metals and many other organic and inorganic compounds that adsorb onto the surface of NPs. While these molecules may contribute beneficially as capping and stabilizing agents, they also introduce variability and uncertainty in functional performance and complicate downstream processing [8]. Current purification techniques, such as centrifugation, filtration and dialysis, are increasingly inefficient at industrial scale due to excessive solvent consumption, long processing times, high operational costs, and contamination risks. Challenges are further amplified in microbially mediated systems where NPs may be formed intracellularly, necessitating additional steps such as cell disruption, enzymatic digestion, and multistage purification. Similarly, e-waste and industrial feedstocks often contain co-existing metals and impurities that co-precipitate during synthesis, making it difficult to achieve high-purity products. As a result, achieving consistent product quality remains a critical barrier to regulatory approval and commercial deployment, highlighting the need for integrated synthesis separation process design rather than post-synthesis purification approaches [244].

### 5.4. Cost and Economic Feasibility

Although waste is often perceived as a low-cost resource, this advantage is frequently offset by high costs associated with preprocessing, purification, energy requirements, and scalability. Several studies have noted that green and waste synthesis routes often have lower yield, slower reaction kinetics and additional treatment steps, increasing production costs per unit of material [245]. For instance, waste PCBs contain a complex mixture of valuable metals often requiring advanced separation and pretreatment techniques, significantly increasing operational costs [178]. Similarly, advanced recycling methods for plastics and other waste streams, while environmentally preferable, remain capital intensive and technologically demanding [136]. With agricultural waste systems, such as fruit peels, the cost of raw materials is reduced; however additional steps are required to improve reaction efficiency and yield. Current studies suggest that WDNP synthesis only achieves economic feasibility when integrated into a biorefinery model where valuable by-products are generated alongside the NPs to offset operational costs. Without such improvements, WDNP methods remain less competitive than conventional approaches, which limits their commercialization [246].

### 5.5. Process Scalability and Standardization

Scaling WDNP synthesis from laboratory to industrial production remains a major challenge. Economic success of NP synthesis depends on maintaining the cost of production below market value, while ensuring consistent quality and high yield [247]. While most sustainable synthesis technologies work well under small, controlled laboratory conditions, they encounter significant barriers at scale. From an engineering perspective, maintaining uniform reaction conditions, including temperature, pH, and mixing, in large reactors is difficult, particularly when processing heterogeneous waste substrates or living organisms. Minor variations can significantly affect NP nucleation and growth dynamics, complicating the scaling-up process [34]. Additional challenges include quality control and safety assessments, which further complicate the commercialization process due to the presence of biological residues and contamination risks, necessitating extensive testing and standardization before deployment [3]. NP properties such as surface charge, which influence biological interactions, are highly sensitive to synthesis conditions, and research has demonstrated that NP stability and aggregation vary significantly under simulated physiological conditions (e.g., gastrointestinal pH), with positively charged NPs often exhibiting higher cytotoxicity than their neutral or negatively charged counterparts [248].

To address these challenges, international standardization of nanotechnology (ISO) was initiated in 2005 with the establishment of the ISO Technical Committee (2009), by academic societies and organizations such as ACS Nano, in which the main aim is to promote responsible and early industrialization of NPs through established testing standards that define the characteristics of material, measurement methods and certification procedures [249].

## 6. Strategic Future Directions

To overcome the limitations outlined above and enable the translation of WDNPs from laboratory studies to real-world applications, future research must adopt integrated, systems-level strategies. Progress will depend on addressing reproducibility, scalability, safety, and economic feasibility simultaneously rather than in isolation. The following priorities outline key directions for advancing WDNP technologies:

1. Standardization through advanced analytical frameworks: Future progress must prioritize analytical methods capable of identifying and controlling sources of variables driving batch-to-batch inconsistency. While variability is widely acknowledged, it is rarely quantified or incorporated into process design. Advanced techniques such as high-resolution spectroscopy, multi-omics profiling, and real-time monitoring can improve understanding of how feedstock composition influences nanoparticle formation. However, their high cost limits widespread adoption. Therefore, there is a need for cost-effective and scalable analytical tools that enable reproducibility across laboratories. Updating international standards to include harmonized sampling protocols and measurement methods will be essential for ensuring cross-study comparability and industrial acceptance [250].
2. Pilot-scale validation and techno-economic integration: Transitioning from proof-of-concept research to pilot-scale studies is an important step in advancing WDNPs into real world applications. Pilot-scale facilities are essential to evaluate a more realistic assessment of synthesis under industrial conditions, where factors such as supply logistics, energy consumption and purification efficiency can be properly evaluated, which are often overlooked in small-scale laboratory studies. Techno-economic analysis (TAE) is equally important, as it provides quantitative insights into the economic feasibility of WDNP production, including capital investment, operational costs, process yields, reagent and energy requirements, and potential revenue streams, offering a comprehensive understanding of whether a given approach is commercially viable. Combining TAE with life-cycle assessment (LCA) provides a comprehensive framework, as it allows simultaneous evaluation of economic feasibility and environmental impact ensuring that proposed “sustainable” synthesis routes do not inadvertently introduce higher energy demands or secondary environmental burdens [251].
3. Integration of machine learning (ML) and artificial intelligence (AI): These are emerging as powerful tools for accelerated discovery, optimization, and standardization [252]. AI-driven models can be used to mine many datasets with synthesis parameters, waste compositions, and NP properties to discover hidden correlations and to predict the optimal conditions for reproducibility, whereas machine learning can be used in image analysis for the assessment of an NP’s shape and size, thereby reducing operator bias and aiding in high-throughput characterization [253]. Beyond synthesis, AI also supports “Safe-by-Design” strategies by predicting biological interactions and toxicity prior to experimental validation. When integrated with TEA and LCA frameworks, AI can support holistic decision-making across environmental, economic, and technical dimensions [254].
  However, ML models generally require large volumes of standardized training data to perform reliably, and predictive accuracy and model transferability are reduced when input data are inconsistent, incomplete or limited. Chou et al. [255] noted that data scarcity remains a key challenge even for conventional NP systems and recommended the adoption of FAIR principles, ensuring that datasets are findable, accessible, interoperable and reusable, to support cross-study comparison and data harmonization. For WDNPs, this challenge is further compounded because feedstock composition can vary with geography, season, source type and processing conditions, adding another layer of inconsistency to model training.
4. Comprehensive safety assessment: Current toxicity assessments of WDNPs remain insufficient, as they are largely based on short-term cytotoxicity assays that do not capture long-term or system-level effects. Future research must adopt multiscale and life-cycle-based evaluation frameworks that account for environmental transformations, chronic exposure, and ecological interactions. Advanced in vitro models, including three-dimensional organoids, combined with in vivo and ecotoxicological studies, are essential to replicate realistic biological complexity and for evaluating the risk related to bioaccumulation and trophic transfer [110]. Furthermore, the integration of omics-based approaches (e.g., toxicogenomics, proteomics) can provide mechanistic insights into NP–cell interactions [256]. A key gap remains in establishing standardized dose–response relationships and accurate dosimetry, particularly under environmentally relevant conditions. Without this, the risk assessment remains highly uncertain and difficult to translate into regulatory frameworks. Ultimately, safety assessment must shift from isolated toxicity testing to predictive, system-level risk evaluation, ensuring that WDNP deployment does not introduce unintended environmental or health risks.
5. Socio-economic considerations and alignment with sustainability goals: Commercial adoption of WDNP technologies depends not only on technical feasibility but also on socio-economic and ethical considerations. For instance, WDNPs sourced from animal and biomedical waste may raise concerns about safety, hygiene and cultural acceptability. Transparent risk communications, clear product labelling, disclosure of materials origin, and provision of scientifically validated safety information are essential for building public trust. In addition, equitable access to WDNP technologies must be considered to avoid the exploitation of low-cost waste resources from vulnerable communities [257]. Aligning WDNP research with United Nations sustainable development goals (SDGs) provide a valuable framework for guiding responsible innovation. WDNPs have strong relevance to SDG 3 (health), SDG 6 (clean water), SDG 9 (innovation), and SDG 12 (responsible production). However, trade-offs must be carefully evaluated to avoid unintended environmental or economic consequences [258].
6. Interdisciplinary collaborations and policy integration: Advancing WDNP technologies requires structured collaborations across disciplines and sectors. Collaboration between academia, industry, regulatory bodies, and non-governmental organizations is essential for developing shared infrastructure, data repositories, and standardized methodologies [259]. Effective integration of waste-derived nanotechnologies requires practical incentives that support circular economy approaches. Targeted incentives by the government, such as subsidies, tax benefits, and funding for pilot-scale infrastructure, can accelerate the adoption of waste-to-nanomaterial technologies and long-term investments. Importantly, collaborating with social scientists and ethicists on research frameworks can improve public engagement, address concerns and ensure ethical aspects are incorporated into technological development. Without such interdisciplinary and policy-driven approaches, WDNP technologies risk remaining confined to academic research rather than achieving meaningful environmental and industrial impact [259].

## 7. Conclusions

The escalating environmental and public health challenges associated with global waste accumulation have driven increasing interest in innovative waste valorization strategies, particularly the synthesis of NPs from diverse waste streams. This comprehensive review critically examines recent innovations and potential applications in the conversion of industrial by-products, agricultural residues, electronic, plastics, and animal-derived materials into functional nanomaterials, demonstrating that waste is not merely an environmental liability but produces highly sophisticated, high-purity reservoirs of metals, minerals and biodegradable materials. Importantly, these nanomaterials do not merely replicate conventionally synthesized materials; they often exhibit enhanced or unique properties due to inherent surface chemistries derived from their precursor sources. As a result, WDNPs have demonstrated broad applicability across environmental remediation, sensing technologies, advanced packaging systems, agriculture and sustainable material systems, highlighting their significant technological and environmental potential.

Despite these advances, this review identifies several critical barriers that continue to limit large-scale implementation. The fundamental challenge lies in the intrinsic heterogeneity of waste streams, which leads to variability in NP composition, size, and functionality, ultimately compromising reproducibility and product consistency. This is further compounded by complex purification requirements, uncertainties surrounding long-term environmental and biological impacts, limited standardization of synthesis and characterization protocols. At a broader systems level, inefficient waste segregation, reliance on energy-intensive synthesis routes, and the risk of secondary environmental burdens raise important questions regarding the overall sustainability of current approaches. Risks associated with uncontrolled release or accumulation of NPs in environmental systems further emphasize the need for comprehensive life-cycle-based assessment. Additionally, existing regulatory frameworks remain inadequate to address the complexity of nanomaterials derived from heterogeneous waste sources, while enforcement gaps continue to hinder responsible waste management. These challenges highlight that translation of WDNPs from laboratory research to real-world applications requires coordinated solutions beyond technical optimization alone.

Looking forward, addressing these challenges will require innovative solutions and a strengthened regulatory framework that promote sustainable practices and environmental accountability. Bridging the gap to industrial application requires collaborative and interdisciplinary approaches and pilot-scale validation, techno-economic evaluation, and scalable processing strategies. The integration of emerging tools such as artificial intelligence and machine learning offers further opportunities to optimize synthesis, improve reproducibility, and support predictive safety assessment. Equally important is the need for interdisciplinary collaboration across materials science, environmental science, toxicology, data science, policy, and industry, supported by clear regulatory frameworks and targeted policy incentives. In conclusion, waste-derived nanomaterials represent a promising pathway toward sustainable materials innovation and circular economy implementation. However, without coordinated advances in standardization, safety assessment, and scalable processing, waste-derived nanomaterials risk remaining an academic concept rather than a transformative industrial solution.

## Figures and Tables

**Table 1:** Comparative overview of nanoparticle synthesis methods.

Synthesis Method	Technique	Underlying Principle	Key Applications	Advantages	Limitations	Ref.
Physical	Ball milling	Mechanical grinding via high-energy impact	Alloys, catalysts, ceramics, energy storage	Simple, low cost, large-scale production	High energy, impurity leaching, contamination from milling media	[19]
	Electro-spinning	Electric field-driven fiber formation from polymer jets	Batteries, drug delivery, environmental remediation	Mimics extracellular matrix, high surface area	Needle clogging, high voltage risk, scaling challenges	[20]
	Evaporation-Condensation	Vaporization of bulk material in an inert gas and rapidly cooled	Metallic nanopowders, alloys; semiconductor	Ultra-high purity, avoids chemical or reducing agents	Extreme energy consumption, low production, high electrical demand	[21]
	Laser Ablation	Pulse-induced vaporization and rapid quenching	Sensors, catalysts, medicine	High purity, chemical and surfactant-free	High operational cost, energy intensive	[22]
	PVD	Vacuum-based thin film deposition	Biomedical, catalysts Optoelectronics	Uniform size, high purity, wear-resistant	Complex procedure, low yield, energy-intensive	[23]
Chemical	Chemical Vapor Deposition	Gas-phase reaction on heated substrates	Semiconductor devices, energy storage, optical	High purity materials and thin films	Toxic gases, vacuum/high-temperature energy	[24]
	Co-precipitation	Multiple ionic species precipitating simultaneously	Magnetic NP, superconducting ceramics, MRI contrast agents	Cost-effective, simple, requires low temperatures	Challenging to achieve narrow size distribution, highly sensitive to pH	[25]
	Micro-emulsion	Synthesis within surfactant- nanoreactors	Catalysts, optoelectronic, theranostics	Exceptional control on size and dispersity	Large volumes of toxic surfactants, complex purification	[26]
	Reduction	Conversion of metal ions via reduction	Antimicrobials, catalysis	Minimal equipment, easy to scale and modify	Toxic by-products, hazardous waste, high environmental risk	[27]
	Sol-Gel	Hydrolysis and condensation of liquid precursors	High-entropy alloys, corrosion-resistant, anti-reflective coatings, sensors	Molecular-level control, low processing temperatures	High precursor costs, significant volume shrinkage/cracking	[28]
	Sono-chemistry	Acoustic cavitation-induced radical formation	Biomedical, environmental remediation	Rapid kinetics, high yield, greener than pure chemical	Equipment erosion, precise control of frequency/power	[29]
Biological	Bacterial	Enzymatic reduction via reductases and electron shuttling	Targeted drug delivery, bio-imaging, clinical diagnostics	Exceptional control over size, low energy consumption	Kinetically slow, biosafety risks	[17]
	Fungal	Utilizes secreted protein and mycelial-based fermentation	Wound healing, catalysis, antifungal therapeutics	High biomass yield and stability, ease of handling in large-scale	High fungal debris require intensive downstream purification	[30]
	Macro- and Microalgal	Chelation and reduction via sulfonated polysaccharides	Biodiesel catalysis, biosensors, wastewater treatment	High biocompatibility, cost effective, highly scalable	High capital costs of equipment, sensitivity to light-flux fluctuations.	[31,32]
	Plant extracts	Synergistic redox reactions where phytochemicals reduce metal ions and provide stabilization	Biomedical, antimicrobial coatings, antioxidant, sensors	Rapid reaction kinetics, high scalability, does not require external stabilizing	Seasonal and geographical variability, batch-to-batch inconsistency in NP size.	[33]
	Yeast	Reduction and stabilization via glutathione and membrane-bound proteins	Quantum dot, sensors, antimicrobial, wastewater treatment	Extremely high tolerance to heavy metal toxicity, fewer toxic by-products	Low recovery efficiency, complex purification steps	[34]

**Table 2:** Recent applications and characteristics of agricultural waste-derived NPs.

Agro-Waste	Type of NPs	Size and Shape	Synthesis Technique	Recovery/Yield	Applications	Advantages	Limitation	Reference
Bamboo leaves	Ag	32–35 nm; spherical	Plant-extract-mediated synthesis	NR	Antibacterial, antioxidant, anticancer	Simple synthesis, multifunctional bioactivity	Ag leaching risk in long-term use	[74]
Banana peel	Fe_3_O_4_	14.8 nm; spherical/elongated	Plant-mediated reduction using FeCl_3_·6H_2_O	NR	Antioxidant, food preservation	Low toxicity, renewable feedstock	Extract variability	[75]
	Nanosilica	68–170 nm; spheroidal	Ash pretreatment, acid leaching, alkaline solubilization	Reported graphically; exact value not stated in text	Nano-biopriming, seed germination, plant growth	Supports *Bacillus subtilis* and improves seedling performance	Soil microbiome effects and field-scale validation required	[70]
Banana peel and date seed	ZnO	50 nm; hexagonal	Aqueous extract-mediated synthesis	NR	Low cytotoxicity, therapeutics agents	Low cost, biocompatible synthesis	Feedstock variability; scale-up and purification	[76]
Chickpea peel	Carbon nanotubes (CNTs)	L: 114 nm; D: 7 nm	Low-temp pyrolysis	20% pyrolyzed C recovery; final CNT yield NR	Bioimaging, Cytocompatibility for cancer cells	High conductivity & fluorescence,	Structural uniformity control	[77]
Citrus tree trimmings	CNPs	50 nm; Spherical	Pyrolysis and nutrient KNO_3_ doping	NR	Foliar fertilizer for *P. vulgaris*	Integrates fertilization and sensing	Field-scale validation limited	[78]
Coconut husk	ZnO	9–14 nm	Alkaline extract synthesis	NR	Photocatalysis	Avoid synthetic stabilizers, photocatalytically active	Alkali impurities	[79]
Coffee husk char	C-based nanofluid	6.24 nm; near spherical	Pyrolysis, soaking, sonication, filtration and centrifugation	NR	Solar thermal conversion	High solar absorption and improved photothermal efficiency	Outdoor testing, corrosion and long-term cycling required	[65]
Corn husk	CoO/Co_3_O_4_ biochar catalyst	2 nm	Cobalt impregnation, reduction and pyrolysis	36.1% biochar/catalyst yield; Co oxide NP yield NR	Hydrogen generation	Highly dispersed cobalt oxide on waste biochar	Activity loss during reuse; leaching and regeneration need study	[66]
Corn stalk shell	Carbon QDs	1.2–3.2 nm	Hydrothermal carbonization	NR	Bioimaging	Strong fluorescence, replaces toxic QDs	Scale-up energy use	[80]
Corn stover	CNC, CNF, LCNC and LCNF	Nanocellulose fiber	Nanocellulose extraction with lignin retention	NR	Pickering emulsions, quercetin delivery	UV protection, stable emulsions and improved bioaccessibility	Food safety, validation and in vivo studies required	[55]
Mushroom substrate	AgNP/ToCNF	34 nm; nanofibers	TEMPO-oxidized CNF with in-situ AgNP synthesis	43.21% CNF yield; final AgNP/ToCNF yield NR	Antibacterial biomaterials	Same waste used as cellulose source	Ag release, in vivo safety and long-term stability required	[71]
Orange peel	Ag	16–95 nm	Extract-mediated bioproduction	NR	Antibacterial, antibiofilm	Low-cost, fast synthesis	Batch variability	[81]
Palm waste	Cellulose	97 ± 8 nm; nanofibers	ScCO_2_ extraction and high-pressure homogenization	NR	Antibacterial, wound healing, skin regeneration	High porosity, biocompatibility	Energy intensive	[73]
Peanut Shell	Cu	Spherical & cubic	Enzyme-mediated Lignin peroxidase	NR	Broad antibacterial	Biocatalytic route offers specificity	Enzyme cost	[82]
Potato peel/ coriander	Ag	64–70 nm; spherical	Biogenic synthesis (aqueous extracts)	NR	Antimicrobial, antioxidant, antitumor	Multi-functional bioactivity	Batch variability	[83]
Rice husk	SiNPs	69–71 nm; spherical	Modified sol-gel synthesis	18% recovery; 99% purity	Maize growth, drought stress mitigation, micronutrient uptake	Improved biomass, gas exchange and nutrient uptake	Higher doses caused mild growth inhibition; field validation required	[69]
Rice straw	CNF/Fe(OH)_3_/CMC hydrogel beads	20–70 nm; beads	Cellulose extraction, mechanical defibrillation and Fe(OH)_3_ incorporation	CNF yield: 43% from raw fibers and 33% from extracted cellulose fibers; final bead yield NR	Phosphate recovery, slow-release fertilizer	Links wastewater remediation with fertilizer reuse	Field validation and long-term nutrient release studies required	[60]
	SiNPs	69–71 nm; spherical	Charring, alkaline extraction and hydrothermal synthesis	NR	Dye degradation, antibacterial activity	Strong photocatalytic and antimicrobial activity	UV dependence, reusability and wastewater testing required	[59]
Sugarcane bagasse	Zn-integrated cellulose NPs	341.5 nm; quasi-spherical	Cellulose extraction, zinc incorporation and acid hydrolysis	67.8 ± 1.3% CNP-Zn yield; cellulose extraction yield 0.56 ± 0.01 g/g dry biomass	Antioxidant, biomedical, cosmetic, plant growth	Multifunctional activity and low hemolysis	Moderate anticancer potency; mechanistic and in vivo studies required	[72]
Tomato pomace, olive pomace, mandarin peel, grape seed	SeNPs	118.9–211.5 nm; round	Pectin-stabilized synthesis with polyphenol functionalization	NR	Nutraceutical delivery, antioxidant activity	Improved biocompatibility and gastrointestinal stability	In vitro only; storage stability depends on polyphenol source	[56]
Watermelon peel	TiO_2_ QDs	7 nm; polycrystalline	Extract-assisted hydrothermal synthesis	NR	Antioxidant; antimicrobial	Easy synthesis	Scale-up remains complex	[84]
Wheat Bran	Arabinoxylan-DNA	150–200 nm; Spherical	Cationic modification and self-assembly	NR	Gene delivery in agrochemicals	Biocompatible	Stability and storage challenges	[85]

Abbreviations: Ag: silver; AgNP: silver nanoparticles; C: carbon; CMC: carboxymethyl cellulose; CNF: cellulose nanofiber; CNC: cellulose nanocrystal; CNPs: carbon nanoparticles; CoO: cobalt(II) oxide; Co_3_O_4_: cobalt(II,III) oxide; Cu: copper; Fe_3_O_4_: iron(II,III) oxide; Fe(OH)_3_: iron(III) hydroxide; KNO_3_: potassium nitrate; LCNC: lignin-containing cellulose nanocrystal; LCNF: lignin-containing cellulose nanofiber; QDs: quantum dots; ScCO_2_: supercritical carbon dioxide; SeNPs: selenium nanoparticles; SiNPs: silica nanoparticles; TiO_2_: titanium dioxide; ToCNF: TEMPO-oxidized cellulose nanofiber; ZnO: zinc oxide; NR: not reported.

**Table 3:** Overview of animal waste into high-value nanomaterials.

Animal Waste	Type of NPs	Size and Shape	Synthesis Technique	Recovery/Yield	Applications	Advantages	Limitations	Reference
Black soldier fly pupal exoskeletons	Chitosan	~235 nm; spherical	STPP ionic gelation	29.0 ± 0.2% chitin extraction yield; final yield NR	Antimicrobial biopolymer	Uses insect waste	Limited bacterial testing	[109]
Buffalo bones	Biogenic HAp NPs	57–423 nm; spherical/bud-like	Hydrothermal treatment and calcination	HA phase purity 84.68 to 88.99%; final product yield NR	Bone defect repair	Improved bone healing in rats	High-temperature process	[107]
Chicken bile	Ag	30–45 nm; spherical	Bile-mediated reduction	NR	Antibacterial, antibiofilm	Multifunctional therapeutic properties	Limited availability and variability, Ag toxicity	[121]
Chicken eggshells	CaO	5–30 nm; Spherical	Thermal calcination (700 °C, 7 h)	NR	Antibacterial, antifungal, heavy metal adsorption	Abundant source, high purity	Energy-intensive, particle aggregation, alkalinity may alter soil/water pH	[112]
	CaO nanorods	50–275 nm; Hexagonal rods	Thermal calcination (900 °C, 1 h)	NR	Photocatalysis, antibacterial, electrochemical catalyst	Tunability enhances catalytic activity	Higher temperature increases energy demand	[110]
	ADA-GEL scaffolds	540–585 nm; irregular	Milling and 3D printing	NR	Bone regeneration, 3D printed bone tissue scaffolds	Improved modulus and cytocompatibility	In vitro studies only	[108]
	N,S-doped CDs	10 nm CDs	Green synthesis	NR	Metronidazole aptasensing	Sensitive contaminant detection	Limited field application due to storage requirements	[114]
Chicken feather	Keratin NP-loaded alginate hydrogel	~243 μm; Porous hydrogel	Keratin NP incorporation into alginate	NR	Dentin regeneration	Injectable; cytocompatible	In vitro only	[122]
Cockle shell	Nanocrystalline gypsum	1–150 nm; plate-like	Acid precipitation	Gypsum purity 98.85 to 99.28%, final product yield NR	Industrial-grade gypsum production	High scalability, low toxicity	Acid handling hazards	[123]
Crab & mussel shells	Precipitated CaCO_3_	15–34 nm; Rod-like	Calcination and dissolution	Vaterite phase 91.2 to 98.9%, final PCC yield NR	Non-toxic vaterite-rich biomaterials	Useful for drug delivery, biocompatible	Phase stability issues	[124]
Donkey dung	AgNP-PLA nanofibers	~335 nm fibers; cylindrical	Green synthesis and sequential blow spinning	NR	Antimicrobial, wound dressings	Low-cost precursor	Social acceptance, hygiene concerns, biosafety considerations	[125]
Fish bone	n-HAp	~19.6 nm; rod-like	Thermal extraction/crushing	NR	Bone tissue engineering, osteo and dental	Composition mimics natural bone mineral, high feedstock	Variability in mineral composition, purification required	[126]
Fish scales	HAp	10–30 nm; plate-like	Bead milling & force-spinning	NR	Biomedical	High crystallinity, biocompatible	Energy intensive	[127]
Mollusk shells	Nanocrystalline CaCO_3_	<500 nm; spheroidal	Ball-milling mechanochemistry	NR	Medical-grade biomaterials	High scalability	High energy demand due to milling	[128]
Oyster shells	Mg-doped HAp NPs	10–41 nm; variable shape	One-pot hydrothermal conversion	>99.5 wt% HAp phase transformation; final product yield NR	Bone tissue engineering	Cytocompatible; osteoinductive	Needs in vivo validation	[106]
	Porous HAp NPs	Porous; surface area 55.7 m^2^/g	Pseudomorphic replacement	87.6% HAp phase transformation; final product yield NR	Humic acid removal, water purification	High surface area; real-water testing	Regeneration and scale-up needed	[113]
Salmon bones	n-HAp	28.7 nm; near spherical	Enzymatic extraction & ball milling	NR	Targeted drug delivery	Safe, mild extraction routes	Enzymatic steps increase cost	[127]
Silk sericin	Sericin-capped AgNPs	48–117 nm; spherical	Green reduction	NR	Antibacterial	Protein capping enhances stability	Ag ecotoxicity concerns, Ag cost limit scalability	[129]
	Au/Ag bimetallic NPs	~10 nm; Spherical	Green co-reduction	NR	Wound healing	Multifunctional therapeutic properties	Metal cost, release of metal ions is an environmental risk	[130]
Shrimp shell	Frankincense-loaded chitosan NPs	80–400 nm; morphology varies	Ionic gelation	22.51% chitosan extraction yield; final NP yield NR	Antibiofilm, antimicrobial	Biocompatible, biodegradable; functional surface groups	Batch variability, allergen risk for shellfish-sensitive users	[131]

Abbreviations: ADA-GEL: alginate dialdehyde-gelatin; Ag: silver; AgNPs: silver nanoparticles; AgNP-PLA: silver nanoparticle-poly(lactic acid); Au: gold; CaCO_3_: calcium carbonate; CaO: calcium oxide; CDs: carbon dots; HAp: hydroxyapatite; Mg: magnesium; N,S-doped CDs: nitrogen and sulfur-doped carbon dots; n-HAp: nano-hydroxyapatite; NPs: nanoparticles; PLA: poly(lactic acid); STPP: sodium tripolyphosphate; NR: not reported.

**Table 4:** Overview of recent plastic waste upcycled into high-value nanomaterials.

Plastic Waste	Type of NPs	Size and Shape	Synthesis Technique	Yield/Recovery	Applications	Advantages	Limitations	Reference
ELV plastic	Flash Graphene	~13.8 nm lateral, ~0.358 nm interlayer	Two-step flash joule heating	19–24% flash graphene yield; theoretical total recovery ~25% from raw plastic	Automotive foam reinforcement	Highly scalable with automotive recycling	Requires polymer purification	[156]
Face masks (PP)	Activated C	2–12 nm; Micro/mesoporous	KOH activation	NR	Supercapacitors	Highly scalable, COVID waste valorization	KOH use, emissions during carbonization	[157]
HDPE plastic bags	Carbon dots	1.0–4.5 nm; graphites	Pyrolysis and sonication	NR	Fe^3+^ sensing in water	Eco-friendly route; water sensing	Multi-step processing	[158]
LDPE/LIBs	NiCo alloy@ carbon nanotubes	NiCo (30 nm) inside CNTs (40–60 nm)	High-temp co-pyrolysis	Li recovery >98%; final NiCo alloy@CNT yield NR	Battery recycling, electro-catalysis	High catalytic activity	High-temperature process, metal recovery complexity	[159]
PET—bottle flakes	Carbon QDs	1.6–5.5 nm; quasi-spherical	Hydro-thermal	Up to 48.13% carbon QD yield	Flame-retardant PET additives	Enhances PET performance; circular reuse	C QD separation and uniformity challenges	[160]
PET—textiles	PET carbon dots	1.6–4.6 nm; spherical	Microwave glycolysis	NR	Fe^3+^ sensing, optoelectronic materials	High fluorescence; rapid synthesis	Glycolysis chemicals required	[161]
PET	Porous carbon	0.5–2 nm; nanosheets	Pyrolysis and activation	32.8% PET-PC yield in activation stage	Zn–I_2_ batteries	High surface area, energy storage	Chemical activation required	[162]
	BNC/PVA-supported Pt NPs	3.2 nm	PET hydrolysis + Pt loading	BNC yield 3.0 mg/mL; Pt-BNC/PVA catalyst synthesis yield 97%	Fuel cell catalyst	Low Pt loading, methanol oxidation activity	Multi-step, uses Pt	[150]
PP	N-doped CNTs	Bamboo-like	Catalytic pyrolysis	NR	Antibiotic degradation	Complete sulfamethoxazole removal in 30 min	High temperature, water chemistry affects activity	[146]
	Porous carbon sheets	0.5–2.0 nm; micropores	Catalytic pyrolysis	NR	Supercapacitor electrodes	High surface area; good capacitance	Acid etching adds chemical burden	[163]
PP and PS	Ni-Fe bimetallic nanocatalyst	40 nm	Catalytic pyrolysis	Approximately 30% CNT yield from PS	Oil and CNTs	Highly scalable	Catalyst biomass-derived, high temperature	[155]
Mixed plastics	Flash graphene	16–27 nm sheets; 4–6 layers	Flash joule heating	Variable; HDPE AC-FG yield 21 to 23%, final mixed-plastic ACDC-tFG yield NR	Graphene upcycling	Ultra-fast synthesis, high conductivity	High energy pulse, requires specialized equipment	[164]
Single-use waste plastic	rGO/Fe_3_O_4_ magnetic nanocomposite	rGO sheets	Pyrolysis, Fe_3_O_4_ loading	Final plastic-derived rGO yield NR; 5.75 g rGO/Fe_3_O_4_ obtained from 6 g rGO	Water purification, supercapacitor	Removes diclofenac/caffeine, 488 F/g capacitance	High temperature, acid treatment	[145]
Recycled PU	Ag-HNT/nHAp	Nanotubes, nanocrystals (<100 nm), Nanopores (169–235 μm)	Ring opening/urethane coupling	BHET 86%; PU prepolymer 90%; final scaffold recovery NR	Bone tissue regeneration scaffolds, antibacterial	Biocompatible, moderate scalability	Complex synthesis, multi-component control, biomedical regulation needed	[165]

Abbreviations: ACDC-tFG: alternating current/direct current-treated turbostratic flash graphene; AC-FG: alternating current-treated flash graphene; Ag-HNT/nHAp: silver-halloysite nanotube/nano-hydroxyapatite; BNC: bacterial nanocellulose; C: carbon; CNTs: carbon nanotubes; ELV: end-of-life vehicle; Fe_3_O_4_: iron oxide/magnetite; HDPE: high-density polyethylene; HNT: halloysite nanotube; KOH: potassium hydroxide; LDPE: low-density polyethylene; LIB: lithium-ion battery; nHAp: nano-hydroxyapatite; Ni-Fe: nickel–iron; NiCo: nickel–cobalt; NPs: nanoparticles; NR: not reported; PET: polyethylene terephthalate; PP: polypropylene; PS: polystyrene; Pt: platinum; PU: polyurethane; PVA: polyvinyl alcohol; QDs: quantum dots; rGO: reduced graphene oxide; Zn–I_2_: zinc–iodine.

**Table 5:** Nanoparticles derived from electronic waste (e-waste), including size, key advantages and limitations.

E-Waste	Type of NPs	Size and Shape	Synthesis Technique	Yield/Recovery	Applications	Advantages	Limitations	Reference
Alkaline batteries	Zn-Mn Oxide	60 nm; cylindrical	Hydrothermal/leaching	57.1 wt% yield for ZnMnO	Photocatalytic BPA degradation	Converts battery waste into catalysts	Mixed metal phases, process complexity limit scalability	[192]
Batteries and sugarcane husk	Ag-MnO_2_/PIn nanocomposite	12.6–18.86 nm; agglomerated nanoflakes	Green Ag synthesis and polyindole incorporation	NR	Antibacterial and anticancer activity	Strong antibacterial and cancer-cell inhibition	In vitro only; no normal-cell or in vivo validation	[177]
CPU—metal flakes	Au-loaded COF catalyst	5–10 nm	Au recovery into synthetic COF	>99% Au captured; COF synthesis yields were 73% for TTF-COF and 77% for TPE-COF	CO_2_ fixation and alkyne carboxylation	Selective Au recovery and reusable catalysis	COF is synthetic; only Au is e-waste-derived	[189]
Dry-cell battery-graphite rods	rGO/Ag nanocomposite	2.5 nm	Modified Hummers method and Ag reduction	NR	As(III) electrochemical sensing	Low detection limit for arsenite	Uses strong chemicals; limited reusability data	[193]
Dead cell phone battery copper foil	CuBTC MOF	Octahedral porous particles	Recovered Cu foil with BTC linker	CuBTC yield: 38% without stirring, 72% with stirring, 88% with HNO_3_ but impure, and 44% by hydrothermal synthesis	Bilirubin sensing	Converts battery Cu into diagnostic sensor	Uses DMF; tested mainly in artificial urine	[194]
Electric furnace dust	ZnO	Flake-like, nanorods	Chemical bath deposition	ZnO purity >98%; 84% of ZnO NPs remained on fabric after washing; final ZnO synthesis yield NR	Antibacterial fabric treatment	Metallurgical dust improves material safety	Potential heavy-metal impurities require purification	[195]
Electric cables	CuO NPs	7–14 nm	Acid dissolution, NaOH precipitation, heating and drying	NR	Potential catalysis, sensing, antimicrobial	Simple synthesis and high CuO purity from copper-rich e-waste	Mainly characterization only, no direct application testing, uses strong acid and base	[187]
	Graphene-Cuprous Oxide	50–400 nm; Spherical	Surfactant chemical co-precipitation	NR	Electrochemical sensing	High-sensitivity electrochemical sensing	Energy intensive	[196]
PCB	Au & TiO_2_	4.15 nm; Spherical	Deposition precipitation with urea	NR	High catalytic activity	Precious metal recovery, excellent catalysis	Costly recovery steps, complex separation	[197]
	Ag	76.91 nm; Spherical	Sodium borohydride chemical bath reduction	NR	Antimicrobial cotton textiles	High antimicrobial efficacy, value-added textiles	Chemical reagents increase ecotoxicity	[198]
	Ag	0.3–0.6 µm; rod-shaped	Hydrothermal leaching and calcination	NR	Antioxidant activity	Good metal recovery	High temperature use, energy intensive	[199]
	Cu	5–50 nm; spheres & rods	Ascorbic acid reduction and ammoniacal precipitation	Approximately 86 wt% Cu NP yield		High conductivity, catalysis and electronics	Requires standardized protocols for industrial use	[200]
	CU/CuO	Cu: 343–460 nm; CuO: 20–31 nm	Ascorbic acid reduction and ammoniacal precipitation	96.7% Cu recovery; final Cu/CuO NP yield NR	Antibacterial and photocatalytic (Rhodamine B)	Potential for wastewater treatment	Size heterogeneity may affect performance consistency	[201]
	Cu & Fe_3_O_4_	10–100 nm; spherical	Hydrometallurgical acid leaching and biological reduction	80% recovery of Fe and Cu metals before NP reduction; final NP yield NR	Antifouling, environmental protection	Recovers multiple metals	Acid leaching produces secondary waste, metal purity variability	[202]
PCBs and *Prosopis juliflora* biomass	Green synthesized CuO NPs	15–25 nm; spherical/irregular	Cu leaching followed by plant-mediated synthesis	NR	DCF, BPA and CBZ removal from water	>90% removal, reusable for five cycles, includes LCA	Lab-scale batch study; acid leaching and calcination required	[188]
Zinc-carbon battery anode	CNPs@MoS_2_ and CNPs@WS_2_	100 nm	Coupling battery-derived CNPs with MoS_2_/WS	NR	Hydrogen evolution reaction	Improves conductivity and electrocatalysis	Only carbon is waste-derived	[184]

Abbreviations: Ag, silver; As(III), arsenite; Au, gold; BPA, bisphenol A; BTC, benzene-1,3,5-tricarboxylate; CBZ, carbamazepine; CNPs, carbon nanoparticles; COF, covalent organic framework; CO_2_, carbon dioxide; Cu, copper; CuBTC, copper benzene-1,3,5-tricarboxylate metal-organic framework; CuO, copper oxide; DCF, diclofenac; DMF, dimethylformamide; Fe_3_O_4_, magnetite; HNO_3_, nitric acid; LCA, life cycle assessment; MOF, metal-organic framework; MoS_2_, molybdenum disulfide; NaOH, sodium hydroxide; NPs, nanoparticles; PCB, printed circuit board; PIn, polyindole; rGO, reduced graphene oxide; TiO_2_, titanium dioxide; TPE-COF, tetraphenylethylene-based covalent organic framework; TTF-COF, tetrathiafulvalene-based covalent organic framework; WS_2_, tungsten disulfide; ZnO, zinc oxide.

**Table 6:** Characteristics and recent applications of NPs synthesized from industrial waste.

Industrial Waste	Type of NPs	Size and Shape	Synthesis Technique	Yield/Recovery	Applications	Advantages	Limitations	Reference
Coal tailings	Si/C	30–60 nm	Planetary ball milling	NR	Potential adsorption, EOR, CCS	Simple top-down conversion	Mostly characterization only	[224]
Fly ash	Pt/TiO_2_	10–15 nm; spherical	Hydrothermal	NR	Industrial dye removal	Improved photocatalysis	Industrial adoption limited by Pt cost	[225]
	Biosilica	20–40 nm; spherical	Alkaline extraction	20.45% biosilica yield; SiO_2_ purity increased to 93.63%	Adsorbents, fillers	Highly scalable, high purity	Chemical extraction generates alkaline effluent	[226]
Iron tailing and Raney nickel waste	NiFe_2_O_4_	80–150 nm; elongated/spherical	Co-precipitation, calcination	NR	Dye degradation, antibacterial activity	Magnetic, reusable, visible-light active	High-temperature calcination	[215]
Oilseed meal	C	24.4–48 nm; spherical	Hydrothermal carbonization	Microsphere yield 87 ± 6%	Antimicrobial; thermal protection of biomolecules	Haemocompatible, strong antimicrobial activity	Limited control over size distribution	[219]
Paper/Pulp Lignin	Lignin polymeric NPs	~150 nm; self-assembled	High-pressure homogenization	HCPT loading 24.2 ± 3.1 wt%; encapsulation efficiency 74.4 ± 2.8%; final NP yield NR	Targeted drug delivery	Biocompatible; antioxidant; tunable surface	Batch variability from lignin source; purification required	[218]
Phosphogypsum	Ca-HAp	50–57 nm	Calcination	NR	Pb removal from wastewater	Strong Pb^2+^ adsorption, scalable	Calcination energy cost	[227]
	Nano-calcite	~48 nm	Precipitation	Product purity 99%; final synthesis yield NR	Cementitious materials, agriculture, drug delivery	Utilizes Ca-rich waste, potential for construction materials	Impurities (e.g., radionuclides) possible	[228]
Red mud	Core–shell nanogel	213–705 nm;	Nanogel encapsulation	NR	Immobilization of As-bearing gypsum sludge	Stabilizes hazardous waste	Complex synthesis, scale-up challenging	[211]
	FeO	20 nm; spherical	Mechanical ball milling	NR	Arsenic removal (>82%)	Abundant waste source	Alkalinity and composition affect reproducibility	[210]
	Fe_3_O_4_/CS	10–30 nm	Ultrasonic co-precipitation, chitosan coating	NR	As(III) removal	Magnetic recovery, 96.73% removal	Acid treatment, real wastewater testing needed	[213]
Sludge-Paper mill	HAp	45.422 nm, porous	Wet chemical precipitation	NR for final HAp yield; calcium extraction from paper sludge ash ~71%	Adsorbent, ion exchange	Low-cost Ca–P source, thermally stable	Variable composition affects purity	[229]
Sludge-Paint	Co-AC	15–35 nm	Co impregnation, pyrolysis	NR; Co content 5.87 wt%	Tetracycline degradation	97% removal in 5 min	PMS required, Co leaching risk	[230]
Sludge-Waste	Fe-doped biochar	10–20 nm, sheet-like	Hydrothermal carbonization	NR	Rhodamine B degradation	High catalytic efficiency	Iron leaching risk, oxidant cost	[231]
Tires	CB	~22 nm; chain-like agglomerates	One-step thermal transformation	~81% yield; 81 g product from 100 g waste tire rubber	Energy storage, sensing, catalysis, pigments, concrete modification	Simple, low-cost, high-yield process; good thermal stability and conductivity	Oxidation level and impurity control remain important	[222]
	C	30–40 nm; spherical	High-temperature pyrolysis	NR	Energy storage; sensing	High carbon yield; removal of sulfur and Zn	Energy-intensive process; emissions control required	[221]
	C nanofibers	425–881 nm; bamboo-shaped	Microwave-assisted pyrolysis	NR	Industrial gas purification, catalytic activity	Rapid synthesis	Catalyst residues, low scalability due to filtration	[223]

Abbreviations: As(III), arsenite; Ca-HAp, calcium hydroxyapatite; CB, carbon black; CCS, carbon capture and storage; Co-AC, cobalt nanoparticle-embedded activated carbon; CS, chitosan; EOR, enhanced oil recovery; Fe_3_O_4_, magnetite; FeO, iron oxide; HAp, hydroxyapatite; HCPT, hydroxycamptothecin; NiFe_2_O_4_, nickel ferrite; NPs, nanoparticles; NR, not reported; Pb^2+^, lead ion; PMS, peroxymonosulfate; Pt/TiO_2_, platinum-modified titanium dioxide; SiO_2_, silicon dioxide; TiO_2_, titanium dioxide; wt%, weight percentage; Zn, zinc.

## Data Availability

No new data were created or analyzed in this study.
